# Navigating Through Innovation in Elderly’s Health: A Scoping Review of Digital Health Interventions

**DOI:** 10.3389/phrs.2024.1607756

**Published:** 2024-12-19

**Authors:** Macarena Hirmas-Adauy, Carla Castillo-Laborde, Camila Awad, Anita Jasmen, Maurizio Mattoli, Xaviera Molina, Andrea Olea, Isabel Matute, Fernando Soto, Paola Rubilar, Oscar Urrejola, Tania Alfaro, María Teresa Abusleme Lama, Sophie Esnouf

**Affiliations:** ^1^ Centro de Epidemiología y Políticas de Salud, Facultad de Medicina Clínica Alemana, Universidad del Desarrollo, Santiago, Chile; ^2^ Independent Research Consulting, Santiago, Chile; ^3^ Centro de Informática Biomédica, Instituto de Ciencias e Innovación en Medicina, Facultad de Medicina Clínica Alemana, Universidad del Desarrollo, Santiago, Chile; ^4^ Escuela de Kinesiología, Facultad de Medicina Clínica Alemana, Universidad del Desarrollo, Santiago, Chile; ^5^ Instituto de Salud Poblacional, Facultad de Medicina, Universidad de Chile, Santiago, Chile; ^6^ Unidad de Salud Pública y Bioetica, Departamento de Formación Transversal en Salud, Facultad de Medicina y Ciencias de la Salud, Universidad Central de Chile, Santiago, Chile

**Keywords:** digital health, elderly, caregiver, reviews, telemedicine

## Abstract

**Objectives:**

Comprehensively map and summarize digital health initiatives for the elderly and caregivers.

**Methods:**

Scoping review between April and May 2022 based on Joanna Briggs methodology. Databases used included PubMed, Cochrane Library, CINAHL Plus, and Web of Science, along with grey literature and hand searches. Two reviewers independently conducted screening and eligibility phases, with a third resolving disagreements. Data were thematically analyzed.

**Results:**

The review included 421 documents. Most documents were published between 2013 and 2022, with a recent increase. Most studies, originating from high-income countries, focused on home applications and were mainly in the testing and validation stages. Telephones and computers were the predominant devices. Health objectives included monitoring, prevention, and treatment, with interventions utilizing directed communication and personal health monitoring for individuals, and telemedicine and decision support for healthcare providers.

**Conclusion:**

Increasing integration of technology in older adults’ lives, along with their increasing proficiency, is driving a significant rise in digital health interventions. Despite this growth, further research in middle- and low-income countries, for caregivers and evaluating effectiveness and feasibility of these technological interventions is needed.

## Introduction

Population aging is a worldwide phenomenon, and it is estimated that by 2050, two billion people will be 60 years of age and older (22% of the population). Eighty percent of them will be living in low- and middle-income countries [1].

Population aging signifies improved living conditions and health status in countries; however, it also presents a series of health and social challenges that need to be addressed. Moreover, due to physical and functional limitations, some elderly individuals require a caregiver, a key figure who provides care, makes decisions, and connects them with the healthcare system [2–4].

When examining equity in healthcare access, disparities become evident as certain populations encounter unmet health needs due to factors such as limited-service availability, administrative and cultural barriers, substandard service quality, high costs, and extended waiting times. The implementation of digital health technologies can partially alleviate these disparities by enhancing the interaction between healthcare providers and the population [5].

Digital health is defined as “the use of information and communication technology (ICT) in support of health and related areas” [5]. Technologies provide concrete opportunities to address health system challenges and offer possibilities to improve access, coverage and quality of services [6]. Their use, especially in low-resource settings, could enable progress toward universal health coverage (UHC), with a more equitable and resource-efficient model of care [7].

Digital health technologies have emerged as a significant area of development and research aimed at addressing various health needs [5]. In 2018, the World Health Assembly formally recognized their potential in advancing towards Universal Health Coverage (UHC). In its resolution [8], the Assembly called upon health ministries to evaluate the use of digital health technologies and prioritize their development, assessment, implementation, scaling up, and increased use. Furthermore, the resolution tasked the World Health Organization (WHO) with providing policy guidance in this field [5].

The utilization of digital health technologies has been extensively acknowledged for its applicability across various target groups, including patients, healthcare professionals, and decision-makers. This technology encompasses a broad range of functionalities such as electronic health records, emergency alert systems, fall detection sensors, and remote patient monitoring, all of which are continually advancing due to the dynamic nature of the field [5].

The SARS-CoV-2 pandemic has notably accelerated the adoption and development of health technologies. However, there is a pressing need for systematic evidence, particularly regarding vulnerable populations like the elderly, to inform effective development and implementation strategies. Seniors possess diverse healthcare needs but frequently encounter difficulties in utilizing these technologies. Therefore, it is crucial to design digital health solutions that are user-friendly and tailored to their specific needs and preferences, ensuring that healthcare services remain accessible, efficient, and effective. Digital health innovations can enhance care quality for seniors by improving service delivery, optimizing data management, and facilitating better communication between patients and healthcare providers [5, 9].

The aim of this scoping review is to comprehensively map and summarize digital health initiatives, strategies, programs, innovations or policies for the elderly or their caregivers.

## Methods

The scoping review was conducted following the Joanna Briggs Institute (JBI) Guidance for conducting systematic scoping reviews [10] and the Preferred Reporting Items for Systematic reviews and Meta-Analyses extension for Scoping Reviews (PRISMA-ScR) [11].

### Review Question

What initiatives, strategies, programs, innovations, or digital health policies are aimed at older adults or their caregivers?

### Inclusion Criteria

The inclusion criteria were formulated based on the components of the Population, Concept, and Context (PCC) framework as outlined by the JBI [10]:

− Population: elderly or their caregivers (formal and informal). Elderly is defined as individuals at a stage of life characterized by biological, psychological, and social changes associated with aging. For this study, the population aged 60 years and older was considered, in accordance with the definition established by the World Health Organization [12].
− Concept: the core topic or phenomenon of interest are digital health initiatives, strategies, programs, innovations, policies, oriented to older people or their caregivers. Digital health is understood as “the use of information and communication technology in support of health and related areas.” They are considered those with which the elderly person or caregiver interacts, or that fulfill the function of being a facilitator in their daily life related to some area of health.
− Context: regarding geographic location and specific settings, the scoping considers initiatives implemented in any country or setting, including hospitals, primary care facilities, community health services, elder care centers and residences, other healthcare services, private homes, and both rural and urban settings.

### Exclusion Criteria

Documents were excluded if they were interviews, opinion letters, or other types that did not contain their own methods and results; related exclusively at other age groups, surgical practices or pathology diagnosis; inquired exclusively into aspects of acceptability (i.e., the degree to which population or specific social groups accept the technology and the factors that increase or decrease the likelihood of their use); contained terms as ICT (which is not the subject of this review: intensive chemotherapy, intracranial tumors, Islamabad Capital Territory, among others); were clinical trial protocols, or only described the technology (or its development) without having been tested or used by elderly or caregivers.

Included terms like ICT (that, in our context, means Information and Communication Technology) with meanings unrelated to this review, such as “intensive chemotherapy,” “intracranial tumors,” or “Islamabad Capital Territory,” were excluded. This approach allowed to comprehensively capture studies aligned with our focus while filtering out unrelated documents using the same acronym.

### Databases and Search Strategy

The following biomedical data sources were consulted: PubMed (NCBI), CINAHL Plus with Full Text (EBSCO), Academic Search Ultimate (EBSCO), Cochrane Library (free access by Ministry of Health, Chile), Rehabilitation and Sports Medicine (EBSCO), Web of Science (Clarivate), Scielo.org and Emerald.

Also, grey literature (manuals, technical documents, scientific conference reports and conference presentations) was reviewed in World Health Organization and Pan American Health Organization.

Finally, a hand search was performed in OpenAire/Explore, Working with Older People, Smart Homecare Technology and TeleHealth, Journal of Assistive Technologies and Quality in Ageing and Older Adults and Google Scholar.

To identify relevant studies, a seasoned biomedical librarian (AJ) and four researchers specialized in public health and epidemiology (XM, MTA, MM, and MHA) conducted a comprehensive literature search following the stages recommended by the JBI [13].

Firstly, an initial pilot study search was conducted in PubMed (MHA and AJ) using keywords and Medical Subject Headings (MeSH) terms associated with “elderly,” “frail,” “senior,” “frail elderly,” “digital health,” “e-Health,” “telemedicine,” “telehealth,” “digital therapeutics,” “virtual medicine,” “information and communication technology,” “ICT,” “silver economy,” “mhealth,” “mobile application.” In this study, MeSH terms were not used in the final search as they did not effectively discriminate between relevant and non-relevant articles. Instead, free terms were utilized in the titles and abstracts of the documents, allowing for a more precise and targeted search. This approach detected a broader range of relevant studies and sought to ensure no pertinent research was overlooked. Using free terms refined the search criteria to better match the specific context and nuances of the research focus.

The search strategy was implemented across additional databases to ensure the results aligned consistently with the research question. Subsequently, the keywords identified from the relevant articles in the initial search were utilized to formulate a comprehensive and planned search strategy (Appendix search strategy).

A language filter was applied to include documents in English, French, Portuguese, and Spanish, and only studies in humans were included. There was no restriction by publication year. Finally, a review of the references of the included documents was conducted.

The search was conducted from 4 to 15 April 2022, for indexed literature, and 25 April to 26 May 2022, for grey literature and hand search. Reference management software was used to organize the reference database. Additionally, the search results were managed in Microsoft Excel v.2108, with separate sheets created for the search and review processes.

The titles and abstracts of the documents obtained from the identification phase were screened, selecting those to be reviewed in full text during the eligibility phase. Each document, at both stages, was reviewed by two independent researchers, and disagreements were resolved by a third reviewer, all from the research team (IM, CCL, AJ, CA, AO, PR, XM, MM, MHA, OU, TA, FS, and SE). Most of the reviewers have extensive experience in public health research, epidemiology, and literature reviews.

**FIGURE 1:**
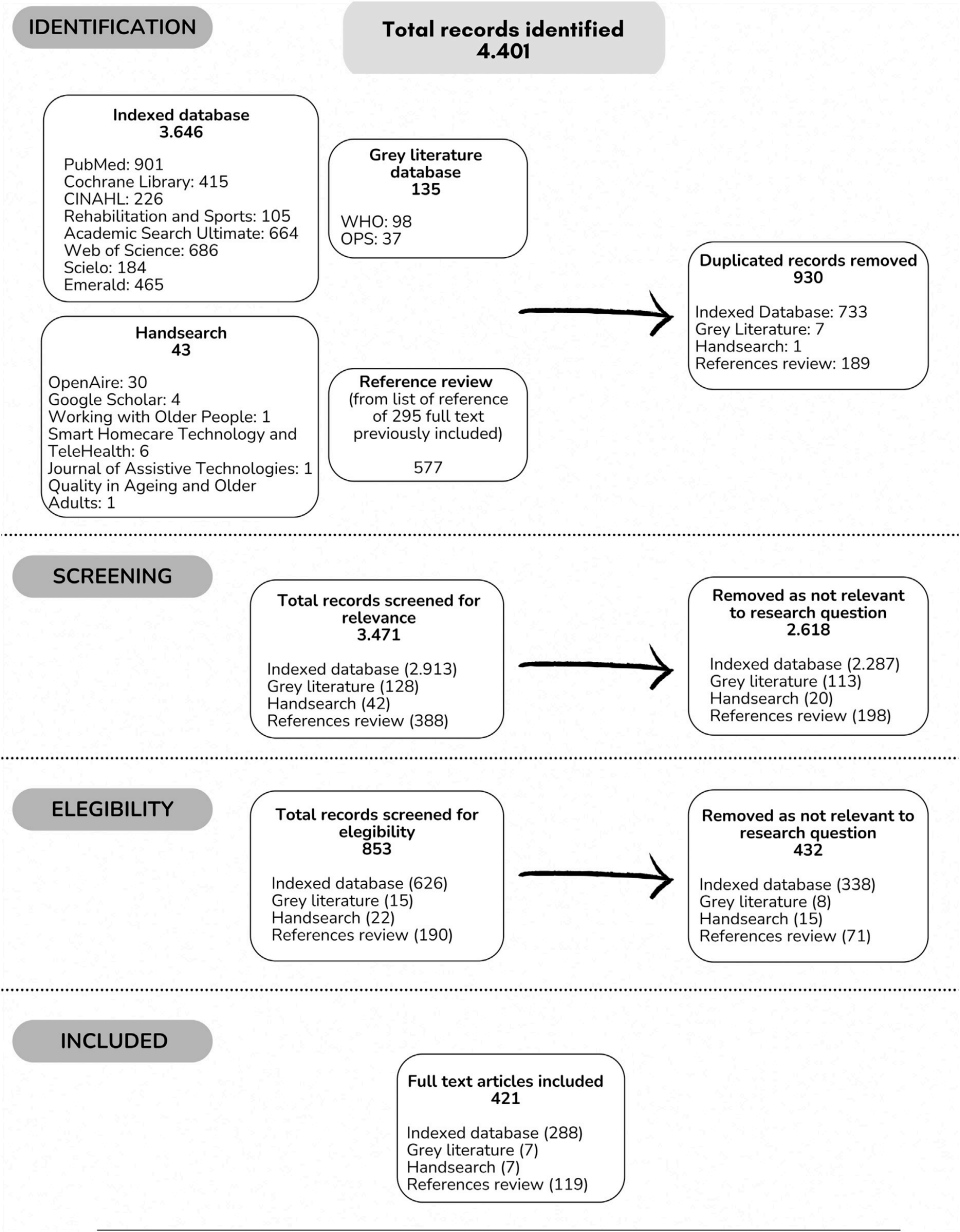
Flowchart of study selection according to scoping review process (Worldwide, 2022).

Full text was searched through open access, by requesting assistance from the Biomedical Library of our university or asking directly from the authors. The entire selection process was diagrammed in a flow chart identifying each stage (identification, screening, and eligibility) (Figure 1).

### Data Extraction

Data extraction from the selected studies followed the methodology outlined by Peters et al. [13]. The research team initially piloted and implemented the extraction process, testing the designed matrix and refining it based on received feedback. This approach facilitated the standardization of criteria for the final data extraction. Upon completion, six researchers (CCL, CA, AO, XM, IM, MHA) reviewed this phase, validating the information extracted from the documents and adjusting as needed.

Data were compiled in a single spreadsheet (Supplementary Material), containing the articles’ main characteristics, along with specific aspects of the review question. Data extraction categories included a detailed overview of each study’s bibliographic information, context, objectives, research design, outcomes, and key findings related to this scoping review [10]. To classify digital health interventions, the WHO’s classification of interventions, services, and applications in health was used [14], and was considered those aimed at person and healthcare providers. Three stages were considered for the level of technological development: *conceptualization and design* (focus on the generation and initial formulation of the intervention, ranging from the initial idea to the design of a functional prototype), *testing and validation* (includes pilot tests and clinical evaluations aimed at testing the intervention in controlled settings or with small groups to validate its efficacy, usability, and safety), and *implementation and scaling* (cover both small and large-scale implementation, commercialization, and post-implementation evaluation, focusing on bringing the intervention into wider use and evaluating its impact and effectiveness in real-world, long-term settings) [15].

### Analysis and Presentation of Results

Data from the selected studies and documents were synthesized by four researchers (CCL, CA, XM, MHA) through an iterative process, according to the research question.

## Results

### Overview

A total of 4,401 records were identified, obtaining 3,471 (78.8%) after eliminating duplicates. Those 3,471 were reviewed in title and abstract (screening). Of them, 853 (24.6%) were selected to continue with the full-text review (eligibility). Finally, 421 (49.4%) documents were included in the analysis and came from: indexed databases 288 (68.4%); grey literature 7 (1.7%); hand search 7 (1.7%) and references review 119 (28.3%) [3, 4, 16–434].

When categorized according to indexed articles or grey literature, 97.6% (n = 411) and 2.4% (n = 10) were identified, respectively. Documents published from 1988 to 2022 (the year the review was in progress), with almost 90% of the publications since 2008 (Table 1).

**TABLE 1:** Summary of characteristics of the included documents (Worldwide, 2022).

Characteristic of included documents (n = 421)*		
Characteristic	N	%
Publication year		
1988–2002	13	3.1
2003–2007	33	7.8
2008–2012	86	20.4
2013–2017	136	32.3
2018–2022	153	36.3
Publication type		
Indexed (n = 411)		
Research article	398	96.8
Congress abstract	8	1.9
Poster	1	0.2
PhD thesis	1	0.2
Special communication	2	0.5
Dissertation	1	0.2
Grey literature (n = 10)		
Technical report	6	60.0
Recommendations	2	20.0
Country report	1	10.0
Position paper	1	10.0
Study design (n = 411)		
Clinical trials, Experimental and Quasi experimental	210	51.1
Report, review or discussion article	33	8.0
Systematic review (with or without metanalisis) and scoping review	27	6.6
Case series	26	6.3
Cross-sectional study	25	6.1
Literature review or Narrative review	25	6.1
Cohort study	17	4.1
Mixed method	15	3.6
Qualitative study	12	2.9
Case-control study	9	2.2
Observational not specified	6	1.5
Other^a^	6	1.5
Corresponding author sex (n = 411)		
Female	194	47.2
Male	217	52.8

*[3, 4, 16–434].

^a^ Indexed articles that could not be classified such as: based case management, retrospective descriptive study, technology model User-Centred Design, assistive technology project.

For the 411 indexed articles, the sex of the corresponding author and the study design were recorded. Grey literature was excluded from this analysis due to the nature of the authorships and the type of scientific communication. In this regard, it was found that 47.3% (n = 194) of the corresponding authors were female. About the study design, 51.1% (n = 210) were clinical trials or experimental and 20.2% (n = 83) were observational (Table 1).

### Population

The interventions were primarily aimed at the elderly population (93.3%, n = 393), followed by healthcare professionals (48.0%, n = 202), and caregivers (33.0%, n = 139). Among the interventions focused on caregivers, the most studied group were family or informal caregivers (52.5%, n = 73) (Table 2).

**TABLE 2:** Summary of characteristics of included documents according to PCC model (Population, Context, Concept) (Worldwide, 2022).

Characteristic of included documents (n = 421)*		
Characteristic	N	%
Population		
Elderly	393	93,3
Healthcare professionals	202	48,0
Caregivers (n = 139)		
Family/Informal	73	52,5
Formal	52	37,4
Not specified	54	38,8
Disease or condition		
Chronic disease (hypertension, diabetes, cancer, obesity, chronic obstructive pulmonary disease, chronic pain, musculoskeletal, etc.)	218	51,8
Fragility (low grip strength, falls, balance disorders, etc.)	114	27,1
Neurological (Alzheimer’s, dementia, etc.)	101	24,0
Mental health (depression, anxiety, social isolation, loneliness, etc.)	75	17,8
Healthy ageing and quality of life	55	13,1
Other^a^	21	5,0
Sensory impairment	8	1,9
Infectious disease	6	1,4
Context		
Region of Country (World Bank Region)		
Europe and Central Asia	149	35,4
North America	114	27,1
East Asia and Pacific	78	18,5
Other^b^	22	5,2
Middle East and North Africa	6	1,4
Latin America and the Caribbean	5	1,2
Sub-Saharan Africa	3	0,7
South Asia	1	0,2
Worldwide	39	9,3
Not specified	4	1,0
Income of Country (World Bank Income)		
High	337	80,0
Upper-middle	28	6,7
Lower-middle	4	1,0
Low	1	0,2
Other^c^	8	1,9
Worldwide	39	9,3
Not specified	4	1,0
Setting		
Home	320	76,0
Nursing home (Retirement home, Community care Veterans’ home, Senior living centers, Day care)	120	28,5
Healthcare facility	111	26,4
Other^d^	13	3,1
Healthcare level (n = 111)		
Primary	71	64,0
Secondary	61	55,0
Tertiary	53	47,7
Concept		
Health objective of the digital tool		
Monitoring or follow-up	265	62,9
Prevention	201	47,7
Therapy or treatment	200	47,5
Promotion	86	20,4
Diagnosis	80	19,0
Rehabilitation	70	16,6
Digital health interventions for persons		
Targeted communication to Persons	259	61,5
Personal health tracking	190	45,1
On demand communication with persons	97	23,0
Person to Person communication	81	19,2
Other^e^	38	9,0
Digital health interventions for healthcare providers		
Telemedicine	239	56,8
Healthcare provider decision support	200	47,5
Prescription and medication management	113	26,8
Referral coordination	78	18,5
Other^f^	13	3,1
Healthcare technologies results		
Positive	366	86,9
No differences	36	8,6
Partially positive	11	2,6
Negative	8	1,9
Type of digital health tools		
Hardware or physical devices		
Telephone (cell phone, smart phone, landline phone)	216	51,3
Desktop or laptop computer	166	39,4
Wearable activity monitors (wrist-worn devices, or step counters)	98	23,3
Tablet	91	21,6
Sensors and positioning system	89	21,1
Video game consoles, exergames, balance board, dance mat, handheld remotes, fitness board, buzz controller, force platforms, cameras with gesture recognition, virtual environment non-immersive and immersive	55	13,1
Telehealth devices (with or without health measurement)	50	11,9
Other^g^	37	8,8
Interactive TV	34	8,1
Robots, social robots, robotic rollators, industrial and service robots, assistive telepresence robot	11	2,6
Radio RX/TX with interaction	2	0,5
Software, platforms		
Videoconference platforms	153	36,3
Text/Audio Messaging (SMS, Chat, etc.)	110	26,1
Digital health portals	91	21,6
Health and fitness apps	81	19,2
Video game	63	15,0
Electronic mail	50	11,9
Digital community or groups	35	8,3
Other^h^	34	8,1

^*^[3, 4, 16–434].

^a^Medication problems, acute illnesses, nutrition and dietary status, specific pathologies, medical and hospital care, and health awareness and education.

^b^Combination of region.

^c^Combination of high/upper-middle and high/upper-middle/lower middle.

^d^Public transportation, hospital at home, community centers, day care center, university, outpatient rehabilitation sports club, welfare centers, Aware Home Research Initiative (AHRI) at the Georgia Institute of Technology is a facility designed to facilitate research, while providing an authentic home environment.

^e^Digital therapeutics based on: therapies based on virtual technology and games, home-based exercise and rehabilitation programs, guided and personalized therapies, interventions based on robotics, emotional support and communication, medical devices and telemedicine applications.

^f^Healthcare provider training, Healthcare provider communication, Person-centered health records, Laboratory and Diagnostics Imaging Management, Scheduling and activity planning for healthcare providers, Person-Centered Health Records, Professional advisory roles.

^g^Communication Devices, Recording Devices and Cameras, Emergency and Alarm Systems, Reminders, Virtual and Augmented Reality Devices, Storage and Playback Devices, Personal Devices.

^h^Records and data management software or platforms, interactive and cognitive assistants, telephone services, websites and online platforms, exercise and educational content.

Twenty documents focused exclusively on interventions for healthcare professionals. These interventions included teleophthalmology, emergency triage, cognitive assessments and development of technologies for care and tele-rehabilitation systems. Other areas addressed include reducing emergency admissions, using wearable devices for health monitoring, supporting caregivers with digital tools, and adapting tele-neuropsychology for the COVID-19 pandemic.

Only five documents focused specifically on caregivers. These studies examined the effectiveness of a platform to enhance caregiver competence, satisfaction, and coping abilities, identified mobile apps for caregivers, and outlined best practices from successful interventions. They also analyzed psychosocial interventions using technology, examined web-based interventions, focusing on their development, delivery, and impact on caregiver health outcomes. Additionally, they explored how technology can improve caregivers’ quality of life.

Chronic diseases were the most studied condition, accounting for 51.8% (n = 218) of the total, with hypertension, diabetes, obesity, chronic obstructive pulmonary disease, chronic pain, and joint issues being the most common. Fragility-related issues, including low grip strength, falls, and balance disorders, were the focus of 27.1% (n = 114) of studies. Neurological conditions, such as Alzheimer’s and dementia were examined in 24.0% (n = 101) of the cases. Mental health including depression, anxiety, social isolation, and loneliness comprised 17.8% (n = 75). Studies on healthy ageing and quality of life made up 13.1% (n = 55) of the total. Less than 2% of the studies focused on sensory impairment and infectious disease, respectively (Table 2).

Other conditions were accounted for in 5.0% (n = 21) of the studies and were related to medication problems, acute illnesses, nutrition and dietary status, specific pathologies, medical and hospital care, health awareness and education.

### Context

The studied countries classified by income and region according to the World Bank [435], are presented in Table 2. Of the total of 421 records, it was possible to classify 378 documents (89.8%), the rest did not specify countries (1.0%, n = 4) or referred to the world level (9.3%, n = 39).

When analyzing income, 80.0% (n = 337) were from high-income countries (HIC), 6.7% (n = 28) from upper-middle-income economies (UMIC), 1.0% (n = 4) from lower-middle-income (LMIC), and only 0.2% (n = 1) from low-income countries (LIC). A 1.9% (n = 8) were a combination of high/upper-middle and high/upper-middle/lower middle.

The most studied regions, accounting for 62.5%, with Europe and Central Asia (35.4%, n = 149), and North America (27.1%, n = 114), followed by East Asia and Pacific (18.5%, n = 78).

It was also studied whether the technology was applied at home, in care centers and residences for the elderly, healthcare facilities or other places. It was found that 76,0% (n = 320) declared a home context, 28.5% (n = 120) care centers and residences for the elderly, 26.4% (n = 111) healthcare facilities and 3.1% (n = 13) others (public transportation, hospital at home, community centers, university, outpatient rehabilitation sports club, welfare centers and a facility designed to research providing an authentic home environment).

Of those who studied a healthcare facility (n = 111), it was found that the level of healthcare corresponded to 64.0% (n = 71) primary, 55.0% (n = 61) secondary and 47.7% (n = 53) tertiary.

### Concept

Despite the diverse methodologies and study designs in the reviewed documents, the reported results related to the use of healthcare technologies were predominantly positive (86.9%, n = 366). Additionally, 11% (n = 47) reported no differences or partially positive results, and only 1.9% (n = 8) negative results (Table 2).

When analyzing the level of technological development, 97.4% (n = 410) of the documents indicated some level. Specifically, 18.8% (n = 79) were at the conceptualization and design stage, 69.1% (n = 291) were in testing and validation, and 25.9% (n = 109) were in the implementation and scalability stage. Documents reporting multiple levels of development were mostly reviews (Table 2).

In relation to the health objective sought by the technological tool, it was found that 62.9% (n = 265) addressed monitoring/follow-up; 47.7% (n = 201) prevention; 47.5% (n = 221) treatment; 20.4% (n = 86) promotion; 19.0% (n = 80) diagnosis, and 16.6% (n = 70) rehabilitation (Table 2).

By classifying digital health interventions, services, and applications in health, of the 421 documents, 61.5% (n = 259) focused on targeted communication to persons; 45.1% (n = 190) on personal health tracking; 23.0% (n = 97) on demand communication with persons; 19.2% (n = 81) on person-to-person communication; and 9.0% (n = 38) on other categories. This last category includes digital therapeutics based on virtual technology and games, home-based exercise and rehabilitation programs, guided and personalized therapies, interventions based on robotics, emotional support and communication, and medical devices and telemedicine applications. Of those interventions aimed at healthcare providers, 56.8% (n = 239) were related to telemedicine; 47.5% (n = 200) to healthcare provider decision support; 26.8% (n = 113) to prescription and medication management; 18.5% (n = 78) to referral coordination and 3.1% (n = 13) to other classifications. The latter category considered healthcare provider training, healthcare provider communication, person-centered health records, laboratory and diagnostics imaging management, scheduling and activity planning for healthcare providers, person-centered health records, professional advisory roles (Table 2).

The type of technology related to hardware and software was analyzed. Regarding hardware, 51.3% (n = 216) referred to telephone (cell phone, smart phone, landline phone); 39.4% (n = 166) to desktop or laptop computer; similar proportions (close to 20%) were found for wearables (wrist-worn devices or step counters); Tablet and sensors and positioning system. Less than 15% was found for video game consoles (exergames, balance board, dance mat), interactive TV, robots (social robots, robotic rollators, assistive telepresence robot), and radio RX/TX with interaction. Telehealth devices (with or without health measurement) were found on 11.9% (n = 50). On the other category was found communication and recording devices, cameras, emergency and alarm systems, reminders, storage and playback devices (Table 2).

In terms of software, it was found that the most widely used was videoconference platforms with 36.6% (n = 153); followed by text/audio messaging with 26.1% (n = 110); digital health portals with 21.6% (n = 91), and less than 20% each of the following: health and fitness apps, video games, email, and digital community or groups. In the other category was found records and data management software or platforms interactive and cognitive assistants telephone services websites and online platforms, exercise and educational content (Table 2).

**FIGURE 2:**
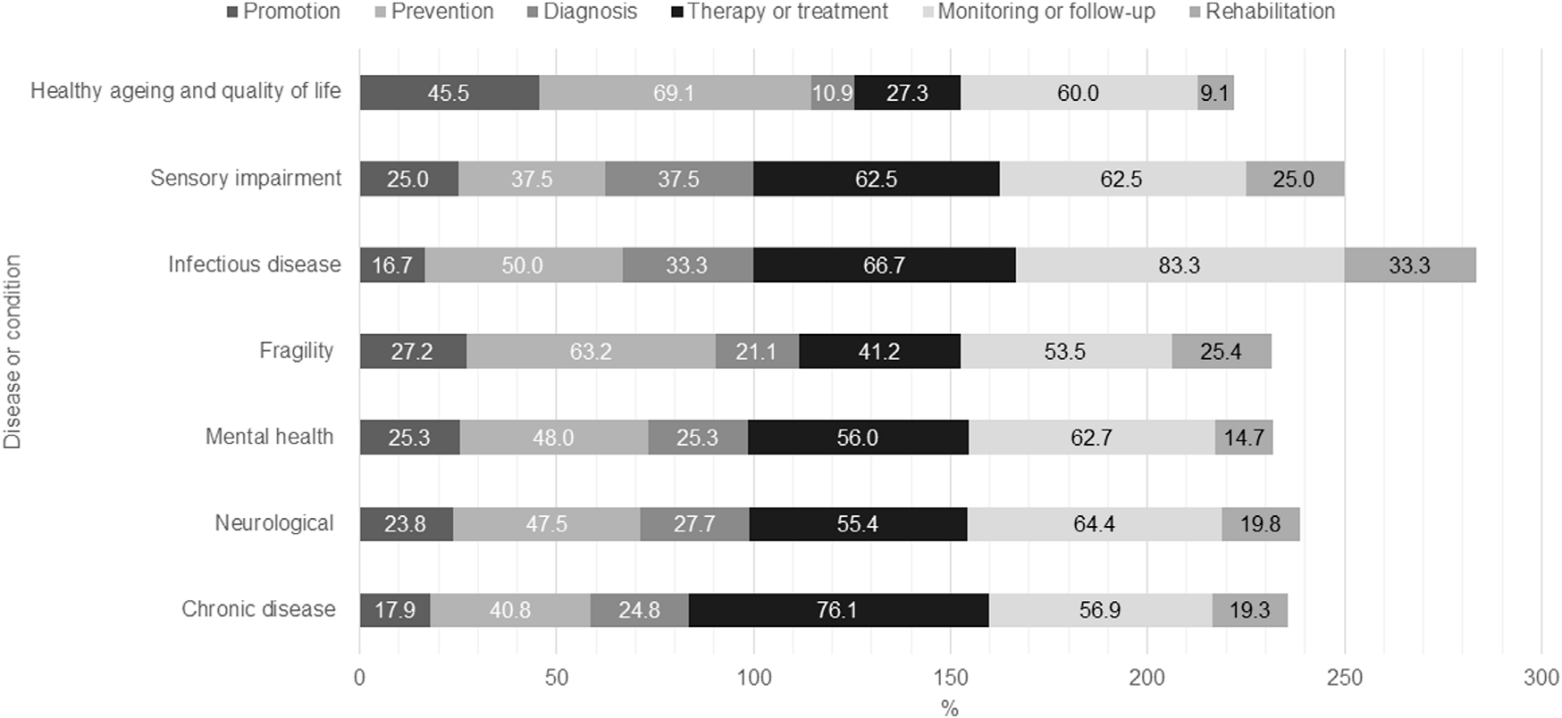
Summary of characteristics of included documents according to Population, Context, Concept (PCC) model (Worldwide, 2022).

In relation to the health objective and the condition under study, for chronic diseases, the most prevalent objective was treatment (76.1%), for fragility was prevention (63.2%), whereas for infectious diseases, it is monitoring/follow-up (83.3%). Meanwhile, prevention and monitoring/follow-up highlight their primary objective in the case of healthy aging and quality of life (69.1% and 60.0%, respectively). The category named other is not described due to the diversity of situations. Figure 2.

**FIGURE 3:**
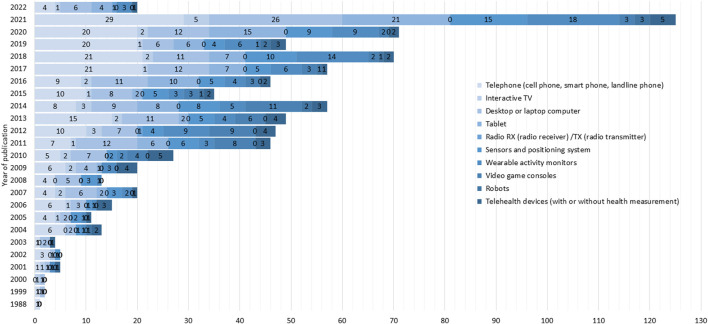
Number of documents by year of publication and type of device studied (Worldwide, 2022).

When the year of publication and type of device studied were analyzed (Figure 3), it was found that over the years, there is a noticeable increase in the use of telephones, especially in the recent period from 2020 to 2022, suggesting a massive adoption of this technology in the analyzed studies (mostly driven by cell phone and smartphone). The use of desktop or laptop computers remains constant over the years, with significant peaks around 2011 and 2022. Activity monitors and sensors show an increasing trend from 2010 onwards, indicating a rise in the analysis of these technologies, particularly in the years 2015, 2017, and 2021. There is a sustained use of telehealth devices over the years, with a notable increase in 2022, possibly reflecting the response to the COVID-19 pandemic and the need for remote health solutions.

**FIGURE 4:**
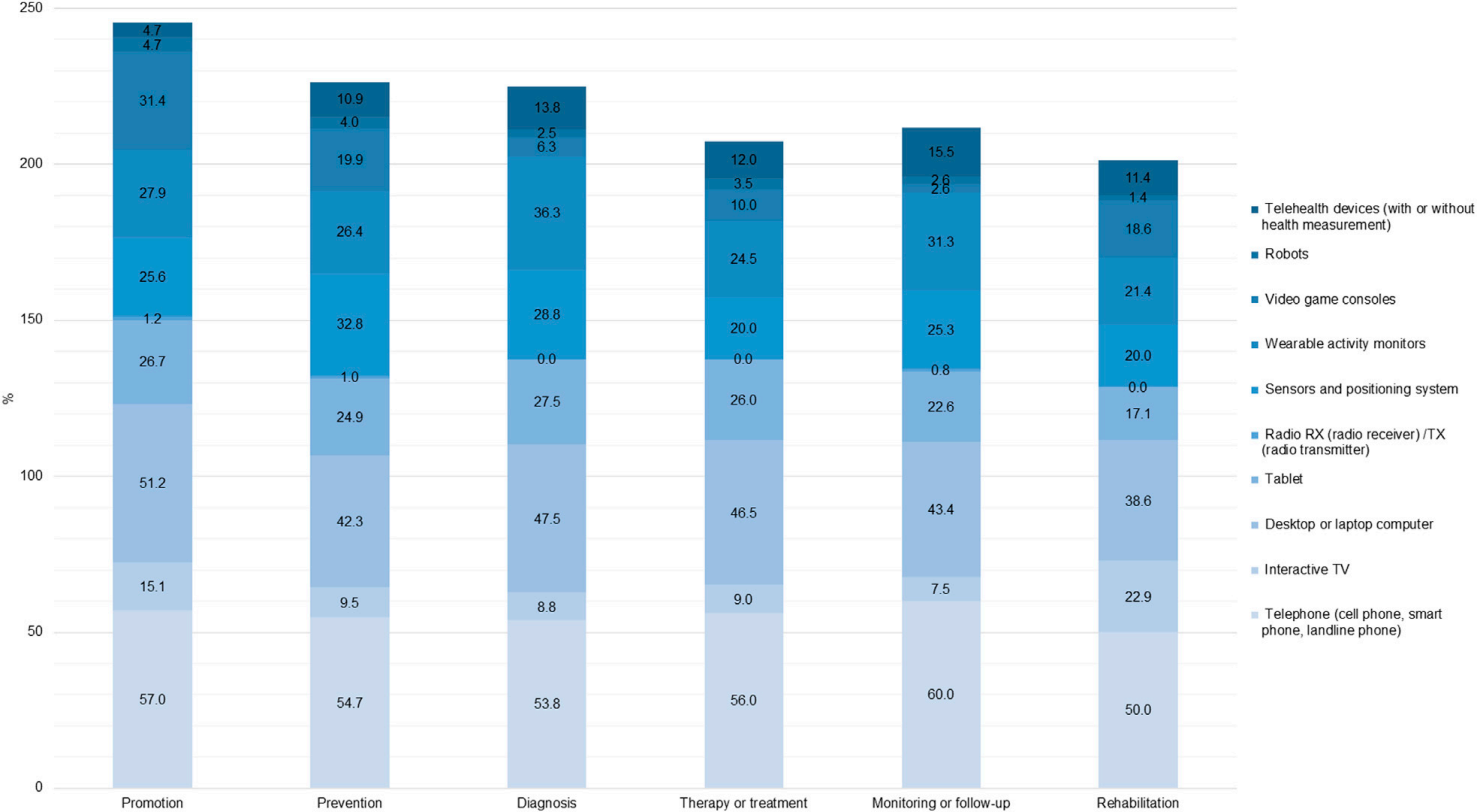
Proportion of documents by health objective and hardware (Worldwide, 2022).

Figure 4 illustrates the utilization of various technological devices across different health objectives, including promotion, prevention, diagnosis, treatment, monitoring/follow-up, and rehabilitation. Telephones and computers are the most used devices for all health objectives analyzed. However, in the case of promotion, video game consoles (31.4%) and wearables (27.9%) also have a significant presence. For prevention and diagnosis, sensors and positioning systems (32.8% and 28.8%, respectively) and wearables (26.4% and 28.8%, respectively) are also prominent. In the case of therapy or treatment, Tablets rank third (26.0%). For monitoring/follow-up, wearable activity monitors (31.3%) and sensors and positioning systems (25.3%) are also considered important, and finally, for rehabilitation, interactive TV (22.9%) is significant. Overall, the figure highlights the diverse roles of these devices in healthcare, with some being more versatile and widely used across multiple functions.

The frequency and distribution of technological devices across multiple diseases, highlights the prominent role of telephones and computers. The data reveals that these devices are extensively used to address chronic diseases (62.8% and 42.2%, respectively), fragility (46.5% and 43.0%) and mental health issues (64.0% and 46.7%). Infectious diseases show same proportion for telephones, computers and Tablets (50.0%, each one). Additionally, telephones show the highest usage for sensory impairments (87.5%). Tablets, wearables and sensors also play a vital role, particularly in neurological conditions (30.7%, 20.8%, and 21.8%) and healthy aging (20.0%, 20.0%, and 25.5%).

## Discussion

There has been an exponential increase in the number of publications on digital health and elderly in recent years. Interventions mainly targeted older adults and healthcare professionals, focusing on chronic conditions, frailty, and neurological diseases. Most studies originated from high-income countries, primarily in Europe and Central Asia, with home being the main application context. Technological development was mostly in the testing and validation stages, with fewer in implementation and scalability. The main objectives were monitoring/follow-up, prevention, and treatment. Interventions focused on directed communication and personal health monitoring for individuals, and telemedicine and decision support for healthcare providers. Throughout the period analyzed, telephones and computers were the most used devices, with videoconferencing and text/audio messaging being the most common software. Over the past decade, there has been an increase in the study of wearables, sensors and positioning systems, and a significant rise in telemedicine in 2022, likely due to the COVID-19 pandemic, highlighting the growing adoption of health technologies and positive trends in reported outcomes.

By 2050, 80% of older people will be living in low- and middle-income countries [1]. Despite this, this study found that most technologies designed to assist the elderly are developed and tested in high-income countries. To enhance access to health services for the elderly, it is important to ensure that these technological solutions are available and accessible where most of this population will reside.

An interesting finding were the context in which the studies were conducted, with 76% stating home as their setting, well above healthcare facilities or elderly residences. This aligns closely with older adults’ preferences to stay in their own homes for as long as possible [436, 437]. In this context, digital health, such as receiving remote care, and preparing homes as smart homes, can become a significant ally in supporting elderly to remain in their homes, helping live as independently as possible.

Most documents indicated some level of development, specifically, in testing and validation. These findings suggest a robust pipeline of technological innovations but also raise questions about their maturity and evaluation. The predominance of projects in the testing and validation phase implies many innovations are still in their early stages and may not have demonstrated efficacy in long-term, real-world environments. This focus on early stages suggests a landscape where technologies are frequently conceptualized and prototyped, but fewer are rigorously evaluated and scaled. It is essential to scrutinize whether these technologies are primarily tested with commercial intentions without thorough evaluations of their long-term impact and effectiveness. Moving forward, it is crucial to emphasize comprehensive evaluation and scalability to ensure technological advancements transition from promising prototypes to impactful solutions that withstand real-world application.

One of the key findings is the high number of interventions targeting chronic diseases focusing on treatment. This indicates a strong emphasis on managing long-term conditions through continuous treatment and monitoring, showcasing the potential of technology to support chronic disease management effectively. For neurological diseases, documents emphasize monitoring/follow-up. This suggests a critical role for technology in tracking disease progression and patient status, which is essential for conditions that require constant observation and timely intervention. Mental health conditions showed a similar trend, focusing on monitoring/follow-up. This highlights the importance of sustained surveillance in mental health to provide timely support and intervention, reflecting the need for persistent engagement and assessment in mental healthcare. When examining interventions for fragility conditions, most documents aimed at prevention. This underscores the proactive approach necessary in addressing issues related to frailty, emphasizing the role of technology in preventing falls, improving balance, and maintaining overall health among the elderly. In the same way, healthy aging and quality of life are more related to prevention, highlights the potential of technology to enhance the quality of life and health outcomes for the aging population, aiming to delay the onset of age-related issues and promote wellness. In any case, all the conditions studied show, to a greater or lesser extent, potential for the use of digital technologies for each of the health objectives. This diversity indicates the multifaceted role of technology in managing any condition, from early detection to comprehensive care and education.

Digital health interventions aimed at treatments, including virtual reality, images, videos, and app prescriptions, do not currently have a corresponding category within the existing WHO framework [14]. However, for those digital interventions with a therapeutic purpose, this classification might need to be reviewed to include a category or type of application or system related to this purpose, likely within the “Services and Application Types” section. Including such a category or type dimension would enhance our understanding of digital health applications’ scope and highlight the potential of emerging technologies to address health treatment and management challenges more effectively with innovative and patient-centered solutions. We have submitted this proposal to WHO through the respective form.

It is important to consider the increasing integration of technology in elderly daily lives, recognizing the growing familiarity and comfort of the aging population with technological tools. As today’s older adults have a greater proficiency with technology, their use of digital health interventions is expected to continue rising [438]. This shift presents opportunities to enhance health outcomes through personalized, accessible, and efficient care. However, it also underscores the need for tailored solutions that address the unique needs and preferences of older adults, ensuring that digital health tools are user-friendly and effectively support their health and wellbeing. Healthcare systems will increasingly need to consider these findings to design and implement effective digital health policies for older adults, overcoming various barriers to accessing healthcare services and digital health for this population group.

The evidence in this area is expanding quickly, not only thanks to rapid advances in technology, but also due to the global aging population and a rising interest in meeting the health and quality-of-life needs of elderly. There is very recent literature on this topic [439–441]; however, these studies primarily focus on specific conditions or isolated contexts. In contrast, this review offers a comprehensive approach, while addressing a wide range of technologies, health conditions and diverse contexts for older adults and their caregivers. This broader scope offers a more comprehensive understanding, addressing key gaps in the existing body of research and providing a crucial foundation for designing interventions and strategies that effectively respond to the complex and interconnected needs of these populations.

However, this study has limitations. Since assessing the quality of the evidence is optional in this type of reviews, low-quality studies are not identified. Detailed information on types of telephones and caregivers was also lacking due to incomplete data in the documents. Additionally, the impact of age is an aspect that warrants special consideration, as it helps to explain the growing number of studies in this area. Elderly individuals are increasingly becoming familiar with technology, which reflects an important shift in their interaction with digital health solutions. Few publications exclusively address caregivers of elderly, even though globally, many people provide care for family members with chronic illnesses, disabilities, or aging-related needs. High-income regions, despite having more developed and institutionalized care systems, also rely on family caregivers [442, 443]. A study found that over 50% of elderly (OECD countries) preferred family care over formal help, influenced by socioeconomic level and cultural values [444]. In Latin America and the Caribbean, the caregiving burden is especially significant, with family members, particularly women, shouldering most responsibilities, often involving extensive unpaid work. This demanding role often results in high levels of stress, burnout, and health issues among caregivers, underscoring the urgent need for enhanced support and resources tailored to family caregivers in these regions [445].

Finally, the high proportion of documents presenting positive results (86.9%), is consistent with the literature trend where positive results are much more represented than negative ones, indicating a potential publication bias [446]. This, among other issues related to underreporting negative results, introduces bias in analyses and misinforms researchers and, consequently, decision-makers.

### Conclusion

Increasing integration of technology in older adults’ lives, along with their increasing proficiency, is driving a significant rise in digital health interventions. Despite this growth, further research in middle- and low-income countries, for caregivers and evaluating effectiveness and feasibility of these technological interventions is needed. The future of digital health for elderly population, caregivers and healthcare professionals presents challenges that must be addressed to realize its full potential. One major issue is accessibility, as these technologies evolve it will be essential ensuring that they are user-friendly and inclusive for all older adults, regardless of their technical skills. Data privacy and security also become critical concerns as more personal health information is shared through digital platforms, demanding robust protections to build and maintain trust among users. Additionally, healthcare providers will face the challenge of integrating digital health tools effectively within traditional care settings, which may require new training and adaptation to evolving workflows. Finally, supporting caregivers and family members in adopting these technologies could be complex, as they often have diverse needs and limited time. Addressing these challenges will be key to harnessing the power of digital health to improve care and quality of life for aging populations.

## Appendix: Search Strategy

**Database:** PubMed (The strategy created for PubMed was modified to suit the requirements of the other databases.)

**Date:** 06 April 2022.

**Key words:** elderly OR frail OR senior OR frail elderly.

**AND**

digital health OR e-Health OR telemedicine OR telehealth OR digital therapeutics OR virtual medicine OR information and communication technology OR ICT OR silver economy OR mhealth OR mobile application.

**Filters:** humans and languages (English, French, Portuguese, Spanish)

**Strategy:**

(“elderly”[Title/Abstract] OR “frail”[Title/Abstract] OR “senior”[Title/Abstract] OR “frail elderly”[Title/Abstract]) AND (“digital health”[Title/Abstract] OR “e-Health”[Title/Abstract] OR “telemedicine”[Title/Abstract] OR “telehealth”[Title/Abstract] OR “digital therapeutics”[Title/Abstract] OR “virtual medicine”[Title/Abstract] OR “ICT”[Title/Abstract] OR “Information and Communication Technology” OR “silver economy”[Title/Abstract] OR “mhealth”[Title/Abstract] OR “mobile application”[Title/Abstract])

## References

[1] World Health Organization. Ageing and Health (2022). Available from: https://www.who.int/news-room/fact-sheets/detail/ageing-and-health (Accessed November 14, 2022).

[2] Riffin C Van Ness PH Iannone L Fried T. Patient and Caregiver Perspectives on Managing Multiple Health Conditions HHS Public Access. J Am Geriatr Soc (2018) 66(10):1992–7. 10.1111/jgs.1550 30153325 PMC6710828

[3] Weiss EF Malik R Santos T Ceide M Cohen J Verghese J Telehealth for the Cognitively Impaired Older Adult and Their Caregivers: Lessons From a Coordinated Approach. Neurodegener Dis Manag (2021) 11(1):83–9. 10.2217/nmt-2020-0041 33172352 PMC7659596

[4] Piette JD Striplin D Marinec N Chen J Trivedi RB Aron DC A Mobile Health Intervention Supporting Heart Failure Patients and Their Informal Caregivers: A Randomized Comparative Effectiveness Trial. J Med Internet Res (2015) 17(6):e142. 10.2196/jmir.4550 26063161 PMC4526929

[5] Organización Mundial de la Salud. WHO Guideline: Recommendations on Digital Interventions for Health System Strengthening (2019). Available from: https://www.who.int/publications/i/item/9789241550505 (Accessed November 14, 2022). 31162915

[6] Rivoir A Morales MJ Casamayou A. Usos y Percepciones de las Tecnologías Digitales en Personas Mayores. Limitaciones y Beneficios para su Calidad de Vida. Revista Austral de Ciencias Sociales (2019) 36:295–313. 10.4206/rev.austral.cienc.soc.2019.n36-15

[7] Evans DB Hsu J Boerma T. Universal Health Coverage and Universal Access. Bull World Health Organ (2013) 91(8):546–A. 10.2471/BLT.13.125450 23940398 PMC3738317

[8] World Health Organization (WHO). The Seventy-First World Health Assembly. Digital health (2018) 4.

[9] Evangelista L Steinhubl SR Topol EJ. Digital Health Care for Older Adults. The Lancet (2019) 393(10180):1493. 10.1016/S0140-6736(19)30800-1 30983579 PMC8106920

[10] Peters M Godfrey C McInerney P Munn Z Tricco A Khalil H. In: Aromataris E Munn Z, editors. Chapter 11: Scoping Reviews (2020 Version). JBI Manual for Evidence Synthesis (2020). Available from: https://synthesismanual.jbi.global (Accessed June 12, 2024).

[11] Tricco AC Lillie E Zarin W O’Brien KK Colquhoun H Levac D PRISMA Extension for Scoping Reviews (PRISMA-ScR): Checklist and Explanation. Ann Intern Med (2018) 169:467–73. 10.7326/M18-0850 30178033

[12] Organización Mundial de la Salud (OMS). Envejecimiento y Salud. World Health Organization (2024). Available from: https://www.who.int/es/news-room/fact-sheets/detail/ageing-and-health (Accessed February 2, 2020).

[13] Peters MDJ Godfrey CM Khalil H McInerney P Parker D Soares CB. Guidance for Conducting Systematic Scoping Reviews. Int J Evid Based Healthc (2015) 13:141–6. 10.1097/XEB.0000000000000050 26134548

[14] World Health Organization. Classification of Digital Interventions, Services and Applications in Health. In: A Shared Language to Describe the Uses of Digital Technology for Health. 2nd ed. (2023). Available from: https://iris.who.int/bitstream/handle/10665/373581/9789240081949-eng.pdf?sequence=1 (Accessed February 29, 2024).

[15] Bhattacharya S Kumar V Narayan NS. Technology Readiness Level: An Assessment of the Usefulness of This Scale for Translational Research. Productivity (2022) 62(2):106–18. 10.32381/PROD.2021.62.02.2

[16] Burdese E Testa M Raucci P Ferreri C Giovannini G Lombardo E Usefulness of a Telemedicine Program in Refractory Older Congestive Heart Failure Patients. Diseases (2018) 6(1):10. 10.3390/diseases6010010 29361704 PMC5871956

[17] Tomita MR Mann WC Stanton K Tomita AD Sundar V Stanton M. Use of Currently Available Smart Home Technology by Frail Elders Process and Outcomes. Top Geriatr Rehabil (2007) 23(1):24–34. 10.1097/00013614-200701000-00005

[18] Blanson Henkemans OA Rogers WA Fisk AD Neerincx MA Lindenberg J, Van Der Mast CAPG. Usability of an Adaptive Computer Assistant That Improves Self-Care and Health Literacy of Older Adults. Methods Inf Med (2008) 47:82–8. 10.3414/me9105 18213433 PMC4209174

[19] Harerimana B Forchuk C O’Regan T. The Use of Technology for Mental Healthcare Delivery Among Older Adults With Depressive Symptoms: A Systematic Literature Review. Int J Ment Health Nurs (2019) 28:657–70. 10.1111/inm.12571 30666762

[20] Kim J Son J Ko N Yoon B. Unsupervised Virtual Reality-Based Exercise Program Improves Hip Muscle Strength and Balance Control in Older Adults: A Pilot Study. Arch Phys Med Rehabil (2013) 94(5):937–43. 10.1016/j.apmr.2012.12.010 23262158

[21] Barrett D. The Role of Telemonitoring in Caring for Older People With Long-Term Conditions. Nurs Old People (2012) 24(7):21–5. 10.7748/nop2012.09.24.7.21.c9257 23008916

[22] Hee CHo G HwanGbo G Soo SHin H. The Effects of Virtual Reality-Based Balance Training on Balance of the Elderly. Phys Ther Sci (2014) 26(4):615–7. 10.1589/jpts.26.615 24764645 PMC3996433

[23] Park EC Kim SG Lee CW. The Effects of Virtual Reality Game Exercise on Balance and Gait of the Elderly. Phys Ther Sci (2015) 27(4):1157–9. 10.1589/jpts.27.1157 25995578 PMC4433999

[24] Larsen LH Schou L Lund HH Langberg H, The Physical Effect of Exergames in Healthy Elderly - A Systematic Review. Games Health J (2013). 2, 205–12. 10.1089/g4h.2013.0036 26192224

[25] Jung H Lee JE. The Impact of Community-Based eHealth Self-Management Intervention Among Elderly Living Alone With Hypertension. J Telemed Telecare (2017) 23(1):167–73. 10.1177/1357633X15621467 26678063

[26] Nakamura K Takano T Akao C. The Effectiveness of Videophones in Home Healthcare for the Elderly. Care (1999) 37:117–25. 10.1097/00005650-199902000-00002 10024116

[27] Bainbridge E Bevans S Keeley B Oriel K. The Effects of the Nintendo Wii Fit on Community-Dwelling Older Adults With Perceived Balance Deficits: A Pilot Study. Phys Occup Ther Geriatr (2011) 29(2):126–35. 10.3109/02703181.2011.569053

[28] Rendon AA Lohman EB Thorpe D Johnson EG Medina E Bradley B. The Effect of Virtual Reality Gaming on Dynamic Balance in Older Adults. Age Ageing (2012) 41(4):549–52. 10.1093/ageing/afs053 22672915

[29] Teixeira E Fonseca H Diniz-Sousa F Veras L Boppre G Oliveira J Wearable Devices for Physical Activity and Healthcare Monitoring in Elderly People: A Critical Review. Geriatrics (Basel) (2021) 6:38, 10.3390/geriatrics6020038 33917104 PMC8167657

[30] Barnett TE Chumbler NR Bruce Vogel W Beyth RJ Qin H Kobb R. The Effectiveness of a Care Coordination Home Telehealth Program for Veterans With Diabetes Mellitus: A 2-Year Follow-Up. Am J Manag Care (2006) 12(8):467–74. 16886889

[31] Franco JR Jacobs K Inzerillo C Kluzik J. The Effect of the Nintendo Wii Fit and Exercise in Improving Balance and Quality of Life in Community Dwelling Elders. Tech Health Care (2012) 20:95–115. 10.3233/THC-2011-0661 22508022

[32] Williams B Doherty NL Bender A Mattox H Tibbs JR. The Effect of Nintendo Wii on Balance: A Pilot Study Supporting the Use of the Wii in Occupational Therapy for the Well Elderly. Occup Ther Health Care (2011) 25(2–3):131–9. 10.3109/07380577.2011.560627 23899030

[33] Kim H Jhoo JH Jang JW. The Effect of Telemedicine on Cognitive Decline in Patients With Dementia. J Telemed Telecare (2017) 23(1):149–54. 10.1177/1357633X15615049 26541171

[34] Guderian B Borreson A Sletten LE Cable K Stecker P Probst MA The Cardiovascular and Metabolic Responses to Wii Fit Video Game Playing in Middle-Aged and Older Adults. Sports Med Phys Fitness (2010) 50:436–42. 21178930

[35] Daniel K. Wii-Hab for Pre-frail Older Adults. Rehabil Nurs (2012) 37(4):195–201. 10.1002/rnj.25 22744992

[36] Toulotte C Toursel C Olivier N. Wii Fit® Training vs. Adapted Physical Activities: Which One Is the Most Appropriate to Improve the Balance of Independent Senior Subjects? A Randomized Controlled Study. Clin Rehabil (2012) 26(9):827–35. 10.1177/0269215511434996 22324055

[37] Wasilewski MB Stinson JN Cameron JI. Web-Based Health Interventions for Family Caregivers of Elderly Individuals: A Scoping Review. Int J Med Inform (2017) 103:109–38. 10.1016/j.ijmedinf.2017.04.009 28550996

[38] Hsieh WM Chen CC Wang SC Tan SY Hwang YS Chen SC Virtual Reality System Based on Kinect for the Elderly in Fall Prevention. Tech Health Care (2014) 22(1):27–36. 10.3233/THC-130769 24361986

[39] Molina KI Ricci NA De Moraes SA Perracini MR. Virtual Reality Using Games for Improving Physical Functioning in Older Adults: A Systematic Review. J NeuroEngineering Rehabil (2014) 11:156. 10.1186/1743-0003-11-156 25399408 PMC4247561

[40] Pietrzak E Cotea C Pullman S. Using Commercial Video Games for Falls Prevention in Older Adults: The Way for the Future? J Geriatr Phys Ther (2014). 37:166–77. 24406711 10.1519/JPT.0b013e3182abe76e

[41] Kruse C Fohn J Wilson N Patlan EN Zipp S Mileski M. Utilization Barriers and Medical Outcomes Commensurate With the Use of Telehealth Among Older Adults: Systematic Review. JMIR Med Inform (2020) 8(8):e20359. 10.2196/20359 32784177 PMC7450384

[42] Chatterjee S Byun J Pottathil A Moore MN Dutta K Qi H Persuasive Sensing: A Novel in-Home Monitoring Technology to Assist Elderly Adult Diabetic Patients. Springer (2012). p. 31–42.

[43] Maust DT Mavandadi S Benson A Streim JE DiFilippo S Snedden T Telephone-Based Care Management for Older Adults Initiated on Psychotropic Medication. Int J Geriatr Psychiatry (2013) 28(4):410–6. 10.1002/gps.3839 22678956 PMC3514587

[44] Brignell M Wootton R Gray L. The Application of Telemedicine to Geriatric Medicine. Age and Ageing (2007) 36:369–74. 10.1093/ageing/afm045 17449535

[45] Salles N Baudon MP Caubet C Dallay F Chaleuil M Magne S Telemedicine Consultations for the Elderly With Chronic Wounds, Especially Pressure Sores. Eur Res Telemed (2013) 2(3–4):93–100. 10.1016/j.eurtel.2013.06.001

[46] Forducey PG Glueckauf RL Bergquist TF Maheu MM Yutsis M. Telehealth for Persons With Severe Functional Disabilities and Their Caregivers: Facilitating Self-Care Management in the Home Setting. Psychol Serv (2012) 9(2):144–62. 10.1037/a0028112 22662729 PMC3375593

[47] Hamilton T Johnson L Quinn BT Coppola J Sachs D Migliaccio J Telehealth Intervention Programs for Seniors: An Observational Study of a Community-Embedded Health Monitoring Initiative. Telemed e-Health (2020) 26(4):438–45. 10.1089/tmj.2018.0248 30994409

[48] Czaja SJ Rubert MP. Telecommunications Technology as an Aid to Family Caregivers of Persons With Dementia. Psychosom Med (2002) 64:469–76. 10.1097/00006842-200205000-00011 12021420

[49] Williams KN Perkhounkova Y Shaw CA Hein M Vidoni ED Coleman CK. Supporting Family Caregivers With Technology for Dementia Home Care: A Randomized Controlled Trial. Innov Aging (2019) 3(3):igz037. 10.1093/geroni/igz037 31660443 PMC6794215

[50] Webster D Celik O. Systematic Review of Kinect Applications in Elderly Care and Stroke Rehabilitation. J NeuroEng Rehabil (2014) 11:108. 10.1186/1743-0003-11-108 24996956 PMC4094409

[51] Kim BYB Lee J. Smart Devices for Older Adults Managing Chronic Disease: A Scoping Review, JMIR Mhealth Uhealth (2017) 5:e69, 10.2196/mhealth.7141 28536089 PMC5461419

[52] Harbig P Barat I Damsgaard EM. Suitability of an Electronic Reminder Device for Measuring Drug Adherence in Elderly Patients With Complex Medication. J Telemed Telecare (2012) 18(6):352–6. 10.1258/jtt.2012.120120 22912488

[53] De Leo D Buono Dello M Dwyer J. Suicide Among the Elderly: The Long-Term Impact of a Telephone Support and Assessment Intervention in Northern Italy. Br J Psychiatry (2002) 181:226–9. 10.1192/bjp.181.3.226 12204927

[54] Keogh JWL Power N Wooller L Lucas P Whatman C. Physical and Psychosocial Function in Residential Aged-Care Elders: Effect of Nintendo Wii Sports Games. J Aging Phys Act (2014) 22(2):235–44. 10.1123/japa.2012-0272 23752164

[55] Regterschot GRH Folkersma M Zhang W Baldus H Stevens M Zijlstra W. Sensitivity of Sensor-Based Sit-To-Stand Peak Power to the Effects of Training Leg Strength, Leg Power and Balance in Older Adults. Gait Posture (2014) 39(1):303–7. 10.1016/j.gaitpost.2013.07.122 23973356

[56] Anderson-Hanley C Snyder AL Nimon JP Arciero PJ. Social Facilitation in Virtual Reality-Enhanced Exercise: Competitiveness Moderates Exercise Effort of Older Adults. Clin Interv Aging (2011) 6(1):275–80. 10.2147/CIA.S25337 22087067 PMC3212419

[57] Kuwahara N Yasuda K Tetsutani N Morimoto K. Remote Assistance for People With Dementia at Home Using Reminiscence Systems and a Schedule Prompter. Int J Comput Healthc (2010) 1:126. 10.1504/ijcih.2010.037458

[58] Vincent C Reinharz D Deaudelin I Garceau M Talbot LR. Public Telesurveillance Service for Frail Elderly Living at Home, Outcomes and Cost Evolution: A Quasi Experimental Design With Two Follow-Ups. Health Qual Life Outcomes (2006) 4:41. 10.1186/1477-7525-4-41 16827929 PMC1562360

[59] Campbell AJ Robertson MC La Grow SJ Kerse NM Sanderson GF Jacobs RJ Randomised Controlled Trial of Prevention of Falls in People Aged > or =75 With Severe Visual Impairment: The VIP Trial. Br Med J (2005) 331(7520):817–20. 10.1136/bmj.38601.447731.55 16183652 PMC1246082

[60] Wollersheim D Merkes M Shields N. Physical and Psychosocial Effects of Wii Video Game Use Among Older Women. Aust J Emerging Tech Soc (2010) 8(2):85–98. Available from: https://www.researchgate.net/publication/232613430 (Accessed June 1, 2023).

[61] Gellis ZD Kenaley B McGinty J Bardelli E Davitt J Ten Have T. Outcomes of a Telehealth Intervention for Homebound Older Adults With Heart or Chronic Respiratory Failure: A Randomized Controlled Trial. Gerontologist (2012) 52(4):541–52. 10.1093/geront/gnr134 22241810

[62] Tetley J Hanson E Clarke A. Older People, Telematics and Care. In: Warnes T Warren L Nolan M editors. Care Services for Later Life: Transformations and Critiques. London, UK: Jessica Kinglsey (2001). p. 243–58.

[63] Sun C Sun L Xi S Zhang H Wang H Feng Y Mobile Phone–Based Telemedicine Practice in Older Chinese Patients With Type 2 Diabetes Mellitus: Randomized Controlled Trial. JMIR Mhealth Uhealth (2019) 7(1):e10664. 10.2196/10664 30609983 PMC6682265

[64] Joe J Demiris G. Older Adults and Mobile Phones for Health: A Review. J Biomed Inform (2013) 46:947–54. 10.1016/j.jbi.2013.06.008 23810858 PMC3836587

[65] Sun R Sosnoff JJ. Novel Sensing Technology in Fall Risk Assessment in Older Adults: A Systematic Review. BMC Geriatr (2018) 18:14. 10.1186/s12877-018-0706-6 29338695 PMC5771008

[66] Quinn CC Khokhar B Weed K Barr E Gruber-Baldini AL. Older Adult Self-Efficacy Study of Mobile Phone Diabetes Management. Diabetes Technol Ther (2015) 17(7):455–61. 10.1089/dia.2014.0341 25692373 PMC4808269

[67] Forsetlund L Bjørndal A Rashidian A Jamtvedt G O’Brien MA Wolf F Continuing Education Meetings and Workshops: Effects on Professional Practice and Health Care Outcomes. Cochrane Database Syst Rev (2009) 2009:CD003030. 10.1002/14651858.CD003030.pub2 19370580 PMC7138253

[68] Chen PY Wei SH Hsieh WL Cheen JR Chen LK Kao CL. Lower Limb Power Rehabilitation (LLPR) Using Interactive Video Game for Improvement of Balance Function in Older People. Arch Gerontol Geriatr (2012) 55(3):677–82. 10.1016/j.archger.2012.05.012 22795360

[69] Kamimura T Ishiwata R Inoue T. Medication Reminder Device for the Elderly Patients With Mild Cognitive Impairment. Am J Alzheimers Dis Other Demen (2012) 27(4):238–42. 10.1177/1533317512450066 22739031 PMC10697399

[70] Center for Technology and Aging. mHealth Technologies: Applications to Benefit Older Adults. 2011.

[71] Tamura T Yonemitsu S Itoh A Oikawa D Kawakami A Higashi Y Is an Entertainment Robot Useful in the Care of Elderly People With Severe Dementia? J Gerontol A Biol Sci Med Sci (2004) 59:83–5. 10.1093/gerona/59.1.m83 14718491

[72] Dear BF Zou J Titov N Lorian C Johnston L Spence J Internet-Delivered Cognitive Behavioural Therapy for Depression: A Feasibility Open Trial for Older Adults. Aust New Zealand J Psychiatry (2013) 47(2):169–76. 10.1177/0004867412466154 23152358

[73] Wu YH Faucounau V De Rotrou J Riguet M Rigaud AS. Intervention Psychosociale Auprès d’Aidants Familiaux de Patients Atteints de la Maladie d’Alzheimer et Technologies de l’Information et de la Communication: Une Revue de la Littérature. Psychol NeuroPsychiatrie du Vieillissement (2009) 7(3):185–92. 19720579 10.1684/pnv.2009.0175

[74] Chopik WJ. The Benefits of Social Technology Use Among Older Adults Are Mediated by Reduced Loneliness. Cyberpsychol Behav Soc Netw (2016) 19(9):551–6. 10.1089/cyber.2016.0151 27541746 PMC5312603

[75] Jorgensen MG Laessoe U Hendriksen C Nielsen OBF Aagaard P. Efficacy of Nintendo Wii Training on Mechanical Leg Muscle Function and Postural Balance in Community-Dwelling Older Adults: A Randomized Controlled Trial. Journals Gerontol - Ser A Biol Sci Med Sci (2013) 68(7):845–52. 10.1093/gerona/gls222 23114461

[76] Siegel C Dorner TE. Information Technologies for Active and Assisted Living—Influences to the Quality of Life of an Ageing Society. Int J Med Inform (2017) 100:32–45. 10.1016/j.ijmedinf.2017.01.012 28241936

[77] Kaye JA Maxwell SA Mattek N Hayes TL Dodge H Pavel M Intelligent Systems for Assessing Aging Changes: Home-Based, Unobtrusive, and Continuous Assessment of Aging. J Gerontol B Psychol Sci Soc Sci (2011) 66(Suppl. 1):i180–i190. 10.1093/geronb/gbq095 21743050 PMC3132763

[78] Cotten SR Anderson WA McCullough BM. Impact of Internet Use on Loneliness and Contact With Others Among Older Adults: Cross-Sectional Analysis. J Med Internet Res (2013) 15(2):e39. 10.2196/jmir.2306 23448864 PMC3636305

[79] Barnason S Zimmerman L Schulz P Tu C. Influence of an Early Recovery Telehealth Intervention on Physical Activity and Functioning After Coronary Artery Bypass Surgery Among Older Adults With High Disease Burden. Heart Lung J Acute Crit Care (2009) 38(6):459–68. 10.1016/j.hrtlng.2009.01.010 19944870 PMC2841300

[80] Sato K Kuroki K Saiki S Nagatomi R. Improving Walking, Muscle Strength, and Balance in the Elderly with an Exergame Using Kinect: A Randomized Controlled Trial. Games Health J (2015) 4(3):161–7. 10.1089/g4h.2014.0057 26182059

[81] Barnason S Zimmerman L Nieveen J Schulz P Miller C Hertzog M Influence of a Symptom Management Telehealth Intervention on Older Adults’ Early Recovery Outcomes After Coronary Artery Bypass Surgery. Heart Lung J Acute Crit Care (2009) 38(5):364–76. 10.1016/j.hrtlng.2009.01.005 19755186 PMC2900787

[82] Trief PM Teresi JA Eimicke JP Shea S Weinstock RS. Improvement in Diabetes Self-Efficacy and Glycaemic Control Using Telemedicine in a Sample of Older, Ethnically Diverse Individuals Who Have Diabetes: The IDEATel Project. Age Ageing (2009) 38(2):219–25. 10.1093/ageing/afn299 19171951

[83] Bernocchi P Vitacca M La Rovere MT Volterrani M Galli T Baratti D Home-Based Telerehabilitation in Older Patients With Chronic Obstructive Pulmonary Disease and Heart Failure: A Randomised Controlled Trial. Age Ageing (2018) 47(1):82–8. 10.1093/ageing/afx146 28985325

[84] Pecina JL Hanson GJ Van Houten H Takahashi PY. Impact of Telemonitoring on Older Adults Health-Related Quality of Life: The Tele-ERA Study. Qual Life Res (2013) 22(9):2315–21. 10.1007/s11136-013-0361-5 23408299

[85] Madigan E Schmotzer BJ Struk CJ DiCarlo CM Kikano G Piña IL Home Health Care With Telemonitoring Improves Health Status for Older Adults With Heart Failure. Home Health Care Serv Q (2013) 32(1):57–74. 10.1080/01621424.2012.755144 23438509 PMC4002284

[86] Ludwig W Wolf KH Duwenkamp C Gusew N Hellrung N Marschollek M Health-Enabling Technologies for the Elderly - An Overview of Services Based on a Literature Review. Comput Methods Programs Biomed (2012) 106(2):70–8. 10.1016/j.cmpb.2011.11.001 22115611

[87] Caljouw SR Vuijk PJ Lamoth C. Exergaming: Interactive Balance Training in Healthy Community-Dwelling Older Adults (2011). Available from: https://www.researchgate.net/publication/51994865 (Accessed August 16, 2023).

[88] Salles N Lafargue A Cressot V Glenisson L Barateau M Thiel E Global Geriatric Evaluation Is Feasible during Interactive Telemedicine in Nursing Homes. Eur Res Telemed (2017) 6(2):59–65. 10.1016/j.eurtel.2017.06.002

[89] Grindrod KA Li M Gates A. Evaluating User Perceptions of Mobile Medication Management Applications With Older Adults: A Usability Study. JMIR Mhealth Uhealth (2014) 2(1):e11. 10.2196/mhealth.3048 25099993 PMC4114457

[90] Van Diest M Lamoth CJ Stegenga J Verkerke GJ Postema K. Exergaming for Balance Training of Elderly: State of the Art and Future Developments. J NeuroEngineering Rehabil (2013) 10:101. 10.1186/1743-0003-10-101 24063521 PMC3851268

[91] Rosenberg D Depp CA Vahia IV Reichstadt J Palmer BW Kerr J Exergames for Subsyndromal Depression in Older Adults: A Pilot Study of a Novel Intervention. Am J Geriatr Psychiatry (2010) 18(3):221–6. 10.1097/JGP.0b013e3181c534b5 20173423 PMC2827817

[92] Brox E Luque LF Evertsen GJ Hernandez JEG. Exergames for Elderly: Social Exergames to Persuade Seniors to Increase Physical Activity. In: 2011 5th International Conference on Pervasive Computing Technologies for Healthcare (PervasiveHealth) and Workshops, Dublin, Ireland, May 23–26, 2011 (IEEE) (2011). p. 546–9.

[93] Upton D Upton P Jones T Jutlla K Brooker D. Evaluation of the Impact of Touch Screen Technology on People With Dementia and Their Caregivers Within Care Home Settings. 2011.

[94] Maseda A Millán-Calenti JC Lorenzo-López L Núñez-Naveira L. Efficacy of a Computerized Cognitive Training Application for Older Adults With and Without Memory Impairments. Aging Clin Exp Res (2013) 25(4):411–9. 10.1007/s40520-013-0070-5 23780693

[95] Rodrigues E Valderramas S Rossetin L Gomes AR. Effects of Video Game Training on the Musculoskeletal Function of Older Adults: A Systematic Review and Meta-Analysis. Top Geriatr Rehabil (2014) 30(4):238–45. 10.1097/tgr.0000000000000040

[96] Hagedorn DK Holm E. Effects of Traditional Physical Training and Visual Computer Feedback Training in Frail Elderly Patients. A Randomized Intervention Study. Eur J Phys Rehabil Med (2010) 46:159–68. 20485221

[97] Geraedts H Zijlstra A Bulstra SK Stevens M Zijlstra W. Effects of Remote Feedback in Home-Based Physical Activity Interventions for Older Adults: A Systematic Review. Patient Educ Couns (2013) 91:14–24. 10.1016/j.pec.2012.10.018 23194823

[98] Maillot P Perrot A Hartley A. Effects of Interactive Physical-Activity Video-Game Training on Physical and Cognitive Function in Older Adults. Psychol Aging (2012) 27(3):589–600. 10.1037/a0026268 22122605

[99] Lai CH Peng CW Chen YL Huang CP Hsiao YL Chen SC. Effects of Interactive Video-Game Based System Exercise on the Balance of the Elderly. Gait Posture (2013) 37(4):511–5. 10.1016/j.gaitpost.2012.09.003 23177921

[100] Muellmann S Forberger S Möllers T Bröring E Zeeb H Pischke CR. Effectiveness of eHealth Interventions for the Promotion of Physical Activity in Older Adults: A Systematic Review. Prev Med (2018) 108:93–110. 10.1016/j.ypmed.2017.12.026 29289643

[101] Changizi M Kaveh MH. Effectiveness of the mHealth Technology in Improvement of Healthy Behaviors in an Elderly Population—A Systematic Review. Mhealth (2017) 3:51. 10.21037/mhealth.2017.08.06 29430455 PMC5803024

[102] Studenski S Perera S Hile E Keller V Spadola-Bogard J Garcia J. Interactive Video Dance Games for Healthy Older Adults. Nutr Health Aging (2010) 14(10):850–2. 10.1007/s12603-010-0119-5 21125204 PMC4895197

[103] Lee S Shin S. Effectiveness of Virtual Reality Using Video Gaming Technology in Elderly Adults With Diabetes Mellitus. Diabetes Technol Ther (2013) 15(6):489–96. 10.1089/dia.2013.0050 23560480

[104] Mulas I Putzu V Asoni G Viale D Mameli I Pau M. Clinical Assessment of Gait and Functional Mobility in Italian Healthy and Cognitively Impaired Older Persons Using Wearable Inertial Sensors. Aging Clin Exp Res (2021) 33(7):1853–64. 10.1007/s40520-020-01715-9 32978750 PMC7518096

[105] Kueider AM Parisi JM Gross AL Rebok GW. Computerized Cognitive Training With Older Adults: A Systematic Review. PLoS One (2012) 7(7):e40588. 10.1371/journal.pone.0040588 22792378 PMC3394709

[106] Botsis T Hartvigsen G. Current Status and Future Perspectives in Telecare for Elderly People Suffering from Chronic Diseases. J Telemed Telecare (2008) 14(4):195–203. 10.1258/jtt.2008.070905 18534954

[107] Tsai HH Tsai YF. Changes in Depressive Symptoms, Social Support, and Loneliness Over 1 Year After a Minimum 3-Month Videoconference Program for Older Nursing Home Residents. J Med Internet Res (2011) 13(4):e93. 10.2196/jmir.1678 22086660 PMC3222194

[108] Ejupi A Brodie M Gschwind YJ Schoene D Lord S Delbaere K. Choice Stepping Reaction Time Test Using Exergame Technology for Fall Risk Assessment in Older People. Annu Int Conf IEEE Eng Med Biol Soc (2014) 2014:6957–60. 10.1109/EMBC.2014.6945228 25571596

[109] Leng FY Yeo D George S Barr C. Comparison of iPad Applications With Traditional Activities Using Person-Centred Care Approach: Impact on Well-Being for Persons With Dementia. Dementia (2014) 13(2):265–73. 10.1177/1471301213494514 24339097

[110] Bateni H. Changes in Balance in Older Adults Based on Use of Physical Therapy vs the Wii Fit Gaming System: A Preliminary Study. Physiotherapy (United Kingdom) (2012) 98(3):211–6. 10.1016/j.physio.2011.02.004 22898577

[111] Banbury A Chamberlain D Nancarrow S Dart J Gray L Parkinson L. Can Videoconferencing Affect Older People’s Engagement and Perception of Their Social Support in Long-Term Conditions Management: A Social Network Analysis From the Telehealth Literacy Project. Health Soc Care Community (2017) 25(3):938–50. 10.1111/hsc.12382 27573127

[112] Jenkins C Amella E Mueller M Moore M Hathaway D. An Internet-Assisted Weight Loss Intervention for Older Overweight and Obese Rural Women A Feasibility Study. Comput Inform Nurs (2016) 34:513–9. 10.1097/CIN.0000000000000275 27392256

[113] Bradley N Poppen W. Assistive Technology, Computers and Internet May Decrease Sense of Isolation for Homebound Elderly and Disabled Persons. Technol Disabil (2003) 15:19–25. 10.3233/tad-2003-15104

[114] Tedesco S Barton J O’Flynn B. A Review of Activity Trackers for Senior Citizens: Research Perspectives, Commercial Landscape and the Role of the Insurance Industry. Sensors (Switzerland); 2017.17, 1277, 10.3390/s17061277 28587188 PMC5492436

[115] Kirk A MacMillan F Rice M Carmichael A. An Exploratory Study Examining the Appropriateness and Potential Benefit of the Nintendo Wii as a Physical Activity Tool in Adults Aged ≥ 55 Years. Interact Comput (2013) 25(1):102–14. 10.1093/iwc/iws004

[116] Lamoth C Caljouw SR Postema K. Active Video Gaming to Improve Balance in the Elderly. Stud Health Technol Inform (2011) 167:159–64. Available from: https://www.researchgate.net/publication/51231122 (Accessed April 11, 2023). 21685660

[117] Cheek P Nikpour L Nowun HD. Aging Well with Smart Technology. Nurs Admin Q (2005) 29:329–38. 10.1097/00006216-200510000-00007 16260997

[118] Piau A Campo E Rumeau P Vellas B Nourhasheim F. Aging Society and Gerontechnology: A Solution for an Independent Living? Nutr Health Aging (2013). 10.1007/s12603-013-0356-5 24402399 PMC12878475

[119] Vaportzis E Martin M Gow AJ. A Tablet for Healthy Ageing: The Effect of a Tablet Computer Training Intervention on Cognitive Abilities in Older Adults. Am J Geriatr Psychiatry (2017) 25(8):841–51. 10.1016/j.jagp.2016.11.015 28082016 PMC5444526

[120] Infante‐Rivard C Krieger M Petitclerc M Baumgarten M. A Telephone Support Service to Reduce Medical Care Use Among the Elderly. J Am Geriatr Soc (1988) 36(4):306–11. 10.1111/j.1532-5415.1988.tb02356.x 3351175

[121] Lee JH Kim H Jhoo H Lee U Kim W Lee Y A Telemedicine System as a Care Modality for Dementia Patients in Korea. Alzheimer Dis Assoc Disord (2000) 14:94–101. 10.1097/00002093-200004000-00007 10850748

[122] Barlow J Singh D Bayer S Curry R. A Systematic Review of the Benefits of Home Telecare for Frail Elderly People and Those With Long-Term Conditions. J Telemed Telecare (2007) 13:172–9. 10.1258/135763307780908058 17565772

[123] Chau JP Lee DT Yu DS Chow A Yu WC Chair S A Feasibility Study to Investigate the Acceptability and Potential Effectiveness of a Telecare Service for Older People With Chronic Obstructive Pulmonary Disease. Int J Med Inform (2012) 81(10):674–82. 10.1016/j.ijmedinf.2012.06.003 22789911

[124] Jerant AF Azari R Martinez C Nesbitt TS. A Randomized Trial of Telenursing to Reduce Hospitalization for Heart Failure: Patient-Centered Outcomes and Nursing Indicators. Home Health Care Serv Q (2003) 22(1):1–20. 10.1300/J027v22n01_01 12749524

[125] Shea S Weinstock RS Teresi JA Palmas W Starren J Cimino JJ A Randomized Trial Comparing Telemedicine Case Management With Usual Care in Older, Ethnically Diverse, Medically Underserved Patients With Diabetes Mellitus: 5 Year Results of the IDEATel Study. J Am Med Inform Assoc (2009) 16(4):446–56. 10.1197/jamia.M3157 19390093 PMC2705246

[126] Shea S Weinstock RS Starren J Teresi J Palmas W Field L A Randomized Trial Comparing Telemedicine Case Management With Usual Care in Older, Ethnically Diverse, Medically Underserved Patients With Diabetes Mellitus. J Am Med Inform Assoc (2006) 13(1):40–51. 10.1197/jamia.M1917 16221935 PMC1380195

[127] Agmon M Perry CK Phelan E Demiris G Nguyen HQ. A Pilot Study of Wii Fit Exergames to Improve Balance in Older Adults. J Geriatr Phys Ther (2011) 34(4):161–7. 10.1519/JPT.0b013e3182191d98 22124415

[128] Schoene D Lord SR Delbaere K Severino C Davies TA Smith ST. A Randomized Controlled Pilot Study of Home-Based Step Training in Older People Using Videogame Technology. PLoS One (2013) 8(3):e57734. 10.1371/journal.pone.0057734 23472104 PMC3589451

[129] Schoene D Lord SR Verhoef P Smith ST. A Novel Video Game-Based Device for Measuring Stepping Performance and Fall Risk in Older People. Arch Phys Med Rehabil (2011) 92(6):947–53. 10.1016/j.apmr.2011.01.012 21549352

[130] Diao Y Lou N Liang S Zhang Y Ning Y Li G A Novel Environment-Adaptive Timed Up and Go Test System for Fall Risk Assessment With Wearable Inertial Sensors. IEEE Sens J (2021) 21(16):18287–97. 10.1109/jsen.2021.3082982

[131] Ohta S Nakamoto’ H Shinagawa Y Tanikawa T. A Health Monitoring System for Elderly People Living Alone. J Telemed Telecare (2001) 8:151–6. 10.1177/1357633X0200800305 12097176

[132] Alm N Dye R Gowans G Campbell J Astell A Ellis M. A Communication Support System for Older People With Dementia. Computer (2007) 40:35–41. 10.1109/mc.2007.153

[133] Pichierri G Murer K De Bruin ED. A Cognitive-Motor Intervention Using a Dance Video Game to Enhance Foot Placement Accuracy and Gait Under Dual Task Conditions in Older Adults: A Randomized Controlled Trial. BMC Geriatr (2012) 12:74. 10.1186/1471-2318-12-74 23241332 PMC3538689

[134] Haines TP Russell T Brauer SG Erwin S Lane P Urry S Effectiveness of a Video-Based Exercise Programme to Reduce Falls and Improve Health-Related Quality of Life Among Older Adults Discharged From Hospital: A Pilot Randomized Controlled Trial. Clin Rehabil (2009) 23(11):973–85. 10.1177/0269215509338998 19675115

[135] World Health Organization. WHO Clinical Consortium on Healthy Ageing 2019. In: Report of Consortium meeting; 21–22 November 2019; Geneva, Switzerland (2019). Available from: http://apps.who.int/bookorders (Accessed June 12, 2024).

[136] World Health Organization. Regional Action Plan on Healthy Ageing in the Western Pacific. 2021.

[137] World Health Organization. Integrated Care for Older People (ICOPE): Guidance for Person-Centred Assessment and Pathways in Primary Care. 2019.

[138] Wambui N. Medical Identification and Sensing Technology for Assisting and E-Health Monitoring Systems for Disabled and Elderly Persons. J Biomed Sustain Healthc Appl (2022) 5:9–17. 10.53759/0088/jbsha202202002

[139] Graham CM Jones N. Impact of IoT on Geriatric Telehealth. Working Old People (2020) 24(3):231–43. 10.1108/wwop-04-2020-0012

[140] Wang CC Chen JJ. Overcoming Technophobia in Poorly-Educated Elderly - the HELPS-Seniors Service Learning Program. Int J Automation Smart Tech (2015) 5(3):173–82. 10.5875/ausmt.v5i3.980

[141] Etchemendy D Baños RM Botella C Castilla D. Programa de Revisión de Vida Apoyado en las Nuevas Tecnologías para las Personas Mayores: Una Aplicación de Tecnologías Positivas. Escr Psicol (2010) 3(2):1–7. 10.24310/espsiescpsi.v3i2.13336

[142] Spinsante S. Home Telehealth in Older Patients With Heart Failure – Costs, Adherence, and Outcomes. Smart Homecare Technol Telehealth (2014) 93:93. 10.2147/shtt.s45318

[143] Giovanni L Frédérique L-G Andrea S Francesco B Clara C Stewart J Can Technology-Based Services Support Long-Term Care Challenges in Home Care? Analysis of Evidence From Social Innovation Good Practices Across the EU: CARICT Project Summary Report. Luxembourg: Publications Office (2012). p. 108.

[144] John O Yadufashije C. E-health Biosensor Platform for Noninvasive Health Monitoring for the Elderly in Low Resource Setting. Int J Biomed Eng Sci (2018) 5(3/4):15–30. 10.5121/ijbes.2018.5402

[145] Knight A Fouyaxis J Jarrad G Beski K Cho G Bidargaddi N. Systems to Harness Digital Footprint to Elucidate and Facilitate Ageing in Place. In: Studies in Health Technology and Informatics. IOS Press (2018). p. 91–101. 10.3233/978-1-61499-845-7-91 29507262

[146] World Health Organization and International Telecommunication Union. Be He@lthy Be Mobile. In: A Handbook on How to Implement mDementia (2021). Available from: http://apps.who.int/bookorders (Accessed June 12, 2024).

[147] Watson P Bearpark T Ling J. The Impact of Rapid Response and Telecare Services on Elderly and Vulnerable Residents. Health Soc Care Community (2021) 29(4):897–904. 10.1111/hsc.13123 32803816

[148] Pergolotti M Deal AM Bryant AL Bennett AV Farley E Covington K Senior Sway: Using a Mobile Application to Measure Fall Risk. J Geriatr Phys Ther (2019) 42(3):E101–7. 10.1519/JPT.0000000000000223 31033583

[149] Santana RF Dantas RV Soares TDS Delphino TM Hercules ABS Leite Junior HMT. Telecuidado para Idosos com Azheimer e seus Cuidadores: Revisão Sistemática/Telecare to Elderly People With Alzheimer and Their Caregivers: Systematic Review. Ciência, Cuidado e Saúde (2018) 17(4). 10.4025/cienccuidsaude.v17i4.41653

[150] Courtney KL Lingler JH Mecca LP Garlock LA Schulz R Dick AW Older Adults’ and Case Managers’ Initial Impressions of Community-Based Telehealth Kiosks. Res Gerontol Nurs (2010) 3(4):235–9. 10.3928/19404921-20100504-03 20509594 PMC2932811

[151] Kang GR Kim IK Kim WJ Lee DY. Promotion of Adequate Exercise for Chronic Disorders’ Elderly Through Paced Music. In: Studies in Health Technology and Informatics. IOS Press (2017). p. 136–40. 10.3233/978-1-61499-830-3-136 29295068

[152] Lind L Carlgren G Karlsson D, Old-and With Severe Heart Failure. Telemonitoring by Using Digital Pen Technology in Specialized Homecare: System Description, Implementation, and Early Results. Comput Inform Nurs (2016). 10.1097/CIN.0000000000000252 27223309

[153] Ogawa EF Harris R Dufour AB Morey MC Bean J. Reliability of Virtual Physical Performance Assessments in Veterans During the COVID-19 Pandemic. Arch Rehabil Res Clin Transl (2021) 3(3):100146. 10.1016/j.arrct.2021.100146 34589696 PMC8463460

[154] Ziegler P Stierand K Bahrs C Ahrend MD. Mid-Term Results After Proximal Humeral Fractures Following Angular Stable Plate Fixation in Elderly Patients - Which Scores Can Be Evaluated by a Telephone-Based Assessment? J Orthop Surg Res (2020) 15(1):6. 10.1186/s13018-019-1536-8 31906989 PMC6945577

[155] Pinero De Plaza MA Beleigoli A Mudd A Tieu M McMillan P Lawless M Not Well Enough to Attend Appointments: Telehealth Versus Health Marginalisation. In: Studies in Health Technology and Informatics. Amsterdam, Netherlands: IOS Press BV (2021). p. 72–9.

[156] Tousignant M Boissy P Corriveau H Moffet H. In Home Telerehabilitation for Older Adults After Discharge From an Acute Hospital or Rehabilitation Unit: A Proof-Of-Concept Study and Costs Estimation. Disabil Rehabil Assist Technol (2006) 1(4):209–16. 10.1080/17483100600776965 19260168

[157] World Health Organization. Be He@lthy Be Mobile: A Handbook on How to Implement mAgeing (2018). Available from: http://apps.who.int/bookorders.

[158] World Health Organization. Age-Friendly Environments in Europe: A Handbook of Domains for Policy Action. Copenhagen, Denmark: Health Organization Regional Office for Europe (2017). Available from: http://www.euro.who.int/pubrequest (Accessed June 12, 2024).

[159] Damant J Knapp M Watters S Freddolino P Ellis M King D. The Impact of ICT Services on Perceptions of the Quality of Life of Older People. J Assist Technol (2013) 7(1):5–21. 10.1108/17549451311313183

[160] Mele C Russo-Spena T Marzullo ML Ruggiero A. Boundary Work in Value Co-Creation Practices: The Mediating Role of Cognitive Assistants. J Serv Manag (2022) 33(2):342–62. 10.1108/josm-10-2020-0381

[161] Marra DE Hamlet KM Bauer RM Bowers D. Validity of Teleneuropsychology for Older Adults in Response to COVID-19: A Systematic and Critical Review. Clin Neuropsychologist (2020) 34:1411–52. 10.1080/13854046.2020.1769192 32519594 PMC8899769

[162] Aily JB Barton CJ Mattiello SM Silva Dde O De Noronha M. Telerehabilitation for Knee Osteoarthritis in Brazil: A Feasibility Study. Int J Telerehabil (2020) 12(2):137–48. 10.5195/ijt.2020.6323 33520101 PMC7757647

[163] Hosteng KR Simmering JE Polgreen LA Cremer JF Segre AM Francis SL Multilevel Mhealth Intervention Increases Physical Activity of Older Adults Living in Retirement Community. J Phys Act Health (2021) 18(7):851–7. 10.1123/jpah.2020-0592 34039774 PMC10170790

[164] Harada ND Dhanani S Elrod M Hahn T Kleinman L Fang M. Feasibility Study of Home Telerehabilitation for Physically Inactive Veterans. J Rehabil Res Dev (2010) 47(5):465–75. 10.1682/jrrd.2009.09.0149 20803390

[165] Tousignant M Marquis N Pagé C Imukuze N Métivier A St-Onge V In-home Telerehabilitation for Older Persons With Chronic Obstructive Pulmonary Disease: A Pilot Study. Int J Telerehabil (2012) 13:15–24. 10.5195/ijt.2012.6083 25945192 PMC4296814

[166] Levy CE Silverman E Jia H Geiss M Omura D. Effects of Physical Therapy Delivery via Home Video Telerehabilitation on Functional and Health-Related Quality of Life Outcomes. J Rehabil Res Dev (2015) 52(3):361–70. 10.1682/JRRD.2014.10.0239 26230650

[167] Moffet H Tousignant M Nadeau S Mérette C Boissy P Corriveau H In-Home Telerehabilitation Compared With Faceto-Face Rehabilitation After Total Knee Arthroplasty: A Noninferiority Randomized Controlled Trial. J Bone Jt Surg - Am (2015) 97(14):1129–41. 10.2106/JBJS.N.01066 26178888

[168] Flynn A Preston E Dennis S Canning CG Allen NE. Home-Based Exercise Monitored With Telehealth Is Feasible and Acceptable Compared to Centre-Based Exercise in Parkinson’s Disease: A Randomised Pilot Study. Clin Rehabil (2021) 35(5):728–39. 10.1177/0269215520976265 33272025

[169] Besnier F Gayda M Nigam A Juneau M Bherer L. Cardiac Rehabilitation During Quarantine in COVID-19 Pandemic: Challenges for Center-Based Programs. Arch Phys Med Rehabil (2020) 101:1835–8. 10.1016/j.apmr.2020.06.004 32599060 PMC7319913

[170] Turner AP Wallin MT Sloan A Maloni H Kane R Martz L Clinical Management of Multiple Sclerosis Through Home Telehealth Monitoring Results of a Pilot Project. Int J MS Care (2013) 15(1):8–14. 10.7224/1537-2073.2012-012 24453757 PMC3883035

[171] Dahlgren K Holzmann MJ Carlsson AC Wändell P Hasselström J Ruge T. The Use of a Swedish Telephone Medical Advice Service by the Elderly–A Population-Based Study. Scand J Prim Health Care (2017) 35(1):98–104. 10.1080/02813432.2017.1288816 28277048 PMC5361425

[172] Hutchinson M Wendt N Smith ST. Trial Implementation of a Telerehabilitation Exercise System in Residential Aged Care. In: Studies in Health Technology and Informatics. IOS Press (2018). p. 62–74. 10.3233/978-1-61499-845-7-62 29507260

[173] Waterson P Eason K Tutt D Dent M. Using HIT to Deliver Integrated Care for the Frail Elderly in the UK: Current Barriers and Future Challenges. In: Work (2012). p. 4490–3. 10.3233/WOR-2012-0750-4490 22317413

[174] Söderlund R. The Role of Information and Communication Technology in Home Services: Telecare Does Not Satisfy the Needs of the Elderly. Health Inform J (2004) 10(2):127–37. 10.1177/1460458204042237

[175] Unbehaun D Taugerbeck S Aal K Vaziri DD Lehmann J Tolmie P Notes of Memories: Fostering Social Interaction, Activity and Reminiscence Through an Interactive Music Exergame Developed for People With Dementia and Their Caregivers. Hum Comput Interact (2021) 36(5–6):439–72. 10.1080/07370024.2020.1746910

[176] Lexis M Everink I Van Der Heide L Spreeuwenberg M Willems C De Witte L. Activity Monitoring Technology to Support Homecare Delivery to Frail and Psychogeriatric Elderly Persons Living at Home Alone. Technol Disabil (2013) 25(3):189–97. 10.3233/tad-130377

[177] Queyroux A Saricassapian B Herzog D Müller K Herafa I Ducoux D Accuracy of Teledentistry for Diagnosing Dental Pathology Using Direct Examination as a Gold Standard: Results of the Tel-E-Dent Study of Older Adults Living in Nursing Homes. J Am Med Dir Assoc (2017) 18(6):528–32. 10.1016/j.jamda.2016.12.082 28236609

[178] Jaana M Sherrard H Paré G. A Prospective Evaluation of Telemonitoring Use by Seniors With Chronic Heart Failure: Adoption, Self-Care, and Empowerment. Health Inform J. (2019) 25(4):1800–14. 10.1177/1460458218799458 30247080

[179] Suh MK Chen CA Woodbridge J Tu MK Kim JI Nahapetian A A Remote Patient Monitoring System for Congestive Heart Failure. J Med Syst (2011). p. 1165–79. 10.1007/s10916-011-9733-y 21611788 PMC3236812

[180] De Luca R Torrisi M Bramanti A Maggio MG Anchesi S Andaloro A A Multidisciplinary Telehealth Approach for Community Dwelling Older Adults. Geriatr Nurs (Minneap) (2021) 42(3):635–42. 10.1016/j.gerinurse.2021.03.015 33823421

[181] Lee DTF Leung DYP Lee IFK Lam LW Lee SWY Chan MWM The Impact of Virtual Wards on Frail Older Patients With Chronic Diseases at Home: A Pre and Post Study. Age Ageing (2014) 43:i4. 10.1093/ageing/afu036.15

[182] Barrett E Burke M Whelan S Santorelli A Oliveira BL Cavallo F Evaluation of a Companion Robot for Individuals With Dementia: Quantitative Findings of the MARIO Project in an Irish Residential Care Setting. J Gerontol Nurs (2019) 47(7):36–45. 10.3928/00989134-20190531-01 31237660

[183] Graham SA Jeste DV Lee EE Wu TC Tu X Kim HC Associations Between Heart Rate Variability Measured With a Wrist-Worn Sensor and Older Adults’ Physical Function: Observational Study. JMIR Mhealth Uhealth (2019) 7(10):e13757. 10.2196/13757 31647469 PMC6913722

[184] Grossman MR Zak DK Zelinski EM. Mobile Apps for Caregivers of Older Adults: Quantitative Content Analysis. JMIR Mhealth Uhealth (2018) 6(7):e162. 10.2196/mhealth.9345 30061093 PMC6090169

[185] Sadasivam RS Luger TM Coley HL Taylor BB Padir T Ritchie CS Robot-Assisted Home Hazard Assessment for Fall Prevention: A Feasibility Study. J Telemed Telecare (2014) 20(1):3–10. 10.1177/1357633X13517350 24352900

[186] Haux R Hein A Kolb G Künemund H Eichelberg M Appell JE Information and Communication Technologies for Promoting and Sustaining Quality of Life, Health and Self-Sufficiency in Ageing Societies-Outcomes of the Lower Saxony Research Network Design of Environments for Ageing (GAL). Inform Health Soc Care (2014) 39(3–4):166–87. 10.3109/17538157.2014.931849 25148556

[187] Hanton CR Kwon YJ Aung T Whittington J High RR Goulding EH Mobile Phone-Based Measures of Activity, Step Count, and Gait Speed: Results From a Study of Older Ambulatory Adults in a Naturalistic Setting. JMIR Mhealth Uhealth (2017) 5(10):e104. 10.2196/mhealth.5090 28974482 PMC5645644

[188] Blixen CE Bramstedt KA Hammel JP Tilley BC. A Pilot Study of Health Education via a Nurse-Run Telephone Self-Management Programme for Elderly People With Osteoarthritis. J Telemed Telecare (2004) 10:44–9. 10.1258/135763304322764194 15006216

[189] Ni Q Hernando ABG de la Cruz IP. The Elderly’s Independent Living in Smart Homes: A Characterization of Activities and Sensing Infrastructure Survey to Facilitate Services Development. Sensors (Switzerland) (2015) 15:11312–62. 10.3390/s150511312 26007717 PMC4481963

[190] Suzuki R Hasegawa T. Evaluation of a One-Dose Package Medication Support System for Community-Based Elderly Patients and Eldercare Facilities. Am J Health-System Pharm (2018) 75(9):e202–12. 10.2146/ajhp170176 29691263

[191] Becker TAC Teixeira CRde S Zanetti ML Pace AE Almeida FA Torquato MT Effects of Supportive Telephone Counseling in the Metabolic Control of Elderly People With Diabetes Mellitus. Rev Bras Enferm (2017) 70(4):704–10. 10.1590/0034-7167-2017-0089 28793098

[192] Van Doorn-Van Atten MN Haveman-Nies A Van Bakel MM Ferry M Franco M De Groot LCPGM Effects of a Multi-Component Nutritional Telemonitoring Intervention on Nutritional Status, Diet Quality, Physical Functioning and Quality of Life of Community-Dwelling Older Adults. Br J Nutr (2018) 119(10):1185–94. 10.1017/S0007114518000843 29759110

[193] Mullen EC. Effect of Telephone Counseling on Physical Activity Among Older Effect of Telephone Counseling on Physical Activity Among Older Adult Cancer Survivors Adult Cancer Survivors. Available from: http://hdl.handle.net/10950/97 (Accessed March 5, 2024).

[194] Chang KC Hung SH Hsueh SJ Chao SF Huang WL Chen HS Development of the Houston–Apollo Model for Older People Living in Remote Areas in Taiwan. Geriatr Gerontol Int (2021) 21(3):299–305. 10.1111/ggi.14130 33527540

[195] Borda A Gilbert C Gray K Prabhu D. In: van den Berg MEL Maeder AJ, editors. Consumer Wearable Information and Health Self Management by Older Adults. IOS Press (2018). 10.3233/978-1-61499-845-7-42 29507259

[196] Steils N Woolham J Fisk M Porteus J Forsyth K. Carers’ Involvement in Telecare Provision by Local Councils for Older People in England: Perspectives of Council Telecare Managers and Stakeholders. Ageing Soc (2021) 41(2):456–75. 10.1017/s0144686x1900120x

[197] Zacharopoulou G Zacharopoulou V Dermatis Z Lazakidou A. Adoption of ICT by Elderly With Hip Fracture After a Fall. Stud Health Technol Inform (2018) 251:191–4. 29968635

[198] Volandes AE Zupanc SN Paasche-Orlow MK Lakin JR Chang Y Burns EA Association of an Advance Care Planning Video and Communication Intervention With Documentation of Advance Care Planning Among Older Adults: A Nonrandomized Controlled Trial. JAMA Netw Open (2022) 5(2):e220354. 10.1001/jamanetworkopen.2022.0354 35201306 PMC8874350

[199] Bendixen RM. Assessment of a Telerehabilitation and a Telehomecare Program for Veterans With Chronic Illnesses. Florida, United States: University of Florida (2006).

[200] Yasini M Marchand G. Adoption and Use of a Mobile Health Application in Older Adults for Cognitive Stimulation. In: Studies in Health Technology and Informatics. IOS Press (2016). p. 13–7. 10.3233/978-1-61499-633-0-13 27071867

[201] Goumopoulos C Papa I Stavrianos A. Development and Evaluation of a Mobile Application Suite for Enhancing the Social Inclusion and Well-Being of Seniors. Informatics (2017) 4(3):15. 10.3390/informatics4030015

[202] Vaziri DD Aal K Gschwind YJ Delbaere K Weibert A Annegarn J Analysis of Effects and Usage Indicators for a ICT-Based Fall Prevention System in Community Dwelling Older Adults. Int J Hum Comput Stud (2017) 106:10–25. 10.1016/j.ijhcs.2017.05.004

[203] Kleinpell RM Avitall B. Integrating Telehealth as a Strategy for Patient Management After Discharge for Cardiac Surgery Results of a Pilot Study. J Cardiovasc Nurs (2007) 22:38–42. 10.1097/00005082-200701000-00006 17224696

[204] Resnick HE Ilagan PR Kaylor MB Mehling D Alwan M. TEAhM-Technologies for Enhancing Access to Health Management: A Pilot Study of Community-Based Telehealth. Telemed e-Health (2012) 18(3):166–74. 10.1089/tmj.2011.0122 22364270

[205] Jarvis MA Chipps J Padmanabhanunni A. “This Phone Saved My Life”: Older Persons Experiences and Appraisals of an mHealth Intervention Aimed at Addressing Loneliness. J Psychol Africa (2019) 29(2):159–66. 10.1080/14330237.2019.1594650

[206] Etchemendy E Baños RM Botella C Castilla D Alcañiz M Rasal P An E-Health Platform for the Elderly Population: The Butler System. Comput Educ (2011) 56(1):275–9. 10.1016/j.compedu.2010.07.022

[207] Zulfiqar AA Hajjam A Talha S Hajjam M Hajjam J Ervé S Telemedicine and Geriatrics in France: Inventory of Experiments. Curr Gerontol Geriatr Res (2018) 2018:9042180. 10.1155/2018/9042180 30310388 PMC6166386

[208] Kang HG Mahoney DF Hoenig H Hirth VA Bonato P Hajjar I *In Situ* Monitoring of Health in Older Adults: Technologies and Issues. J Am Geriatr Soc (2010) 58:1579–86. 10.1111/j.1532-5415.2010.02959.x 20646105

[209] Parfitt J Wan A Darzi A Parfitt J Shafi A de Caux R The Use of Wireless Telemedicine Equipment in the Senior Assessment of Surgical Patients in an Accident and Emergency Department. J Telemed Telecare (2001) 7:82. 10.1177/1357633x010070s140 11331045

[210] Yoshida K Yamaoka Y Eguchi Y Sato D Iiboshi K Kishimoto M Remote Neuropsychological Assessment of Elderly Japanese Population Using the Alzheimer’s Disease Assessment Scale: A Validation Study. J Telemed Telecare (2020) 26(7–8):482–7. 10.1177/1357633X19845278 31068063

[211] Geraedts HAE Dijkstra H Zhang W Ibarra F Far IK Zijlstra W Effectiveness of an Individually Tailored Home-Based Exercise Rogramme for Pre-Frail Older Adults, Driven by a Tablet Application and Mobility Monitoring: A Pilot Study. Eur Rev Aging Phys Activity (2021) 18(1):10. 10.1186/s11556-021-00264-y 34154524 PMC8215778

[212] Mshali H Lemlouma T Moloney M Magoni D. A Survey on Health Monitoring Systems for Health Smart Homes. Int J Ind Ergon (2018) 66:26–56. 10.1016/j.ergon.2018.02.002

[213] Choi SD Guo L Kang D Xiong S. Exergame Technology and Interactive Interventions for Elderly Fall Prevention: A Systematic Literature Review. Appl Ergon (2017) 65:570–81. 10.1016/j.apergo.2016.10.013 27825723

[214] Werner C Moustris GP Tzafestas CS Hauer K. User-Oriented Evaluation of a Robotic Rollator That Provides Navigation Assistance in Frail Older Adults With and Without Cognitive Impairment. Gerontology (2018) 64(3):278–90. 10.1159/000484663 29183035

[215] Piette JD Striplin D Marinec N Chen J Aikens JE. A Randomized Trial of Mobile Health Support for Heart Failure Patients and Their Informal Caregivers Impacts on Caregiver-Reported Outcomes. Med Care (2015) 53:692–9. 10.1097/MLR.0000000000000378 26125415 PMC4503477

[216] Sparsa A Doffoel-Hantz V Bonnetblanc JM. Assessment of Tele-Expertise Among Elderly Subjects in Retirement Homes (EHPAD). Ann Dermatol Venereol (2013) 140(3):165–9. 10.1016/j.annder.2012.11.008 23466148

[217] Shahbazi M Bagherian H Sattari M Saghaeiannejad-Isfahani S. The Opportunities and Challenges of Using Mobile Health in Elderly Self-Care. J Educ Health Promot (2021) 10(1):80. 10.4103/jehp.jehp_871_20 34084827 PMC8057191

[218] Intawong K Boonchieng W Lerttrakarnnon P Boonchieng E Puritat K. A- SA SOS: A Mobile and IoT-Based Pre-Hospital Emergency Service for the Elderly and Village Health Volunteers. Int J Adv Comput Sci Appl (2021) 12(4):513–8. 10.14569/ijacsa.2021.0120465

[219] Tindall LR Huebner RA Stemple JC Kleinert HL. Videophone-Delivered Voice Therapy: A Comparative Analysis of Outcomes to Traditional Delivery for Adults With Parkinson’s Disease. Telemed e-Health (2008) 14(10):1070–7. 10.1089/tmj.2008.0040 19119829

[220] Nancarrow S Banbury A Buckley J. Evaluation of a National Broadband Network-Enabled Telehealth Trial for Older People With Chronic Disease. Aust Health Rev (2016) 40(6):641–8. 10.1071/AH15201 27028234

[221] Grossi G Lanzarotti R Napoletano P Noceti N Odone F. Positive Technology for Elderly Well-Being: A Review. Pattern Recognit Lett (2020) 137:61–70. 10.1016/j.patrec.2019.03.016

[222] Reading Turchioe M Grossman LV Baik D Lee CS Maurer MS Goyal P Older Adults Can Successfully Monitor Symptoms Using an Inclusively Designed Mobile Application. J Am Geriatr Soc (2020) 68(6):1313–8. 10.1111/jgs.16403 32157679 PMC7323569

[223] Whitehouse CR Long JA Maloney LM Daniels K Horowitz DA Bowles KH. Feasibility of Diabetes Self-Management Telehealth Education for Older Adults During Transitions in Care. Res Gerontol Nurs (2020) 13(3):138–45. 10.3928/19404921-20191210-03 31834415 PMC8008169

[224] Zhou S Ogihara A Nishimura S Jin Q. Analyzing the Changes of Health Condition and Social Capital of Elderly People Using Wearable Devices. Health Inf Sci Syst (2018) 6(1):4. 10.1007/s13755-018-0044-2 29692887 PMC5910377

[225] Hsu MH Chu TB Yen JC Chiu WT Yeh GC Chen TJ Development and Implementation of a National Telehealth Project for Long-Term Care: A Preliminary Study. Comput Methods Programs Biomed (2010) 97(3):286–92. 10.1016/j.cmpb.2009.12.008 20092907

[226] Kim HH Cho NB Kim J Kim KM Kang M Choi Y Implementation of a Home-Based Mhealth App Intervention Program With Human Mediation for Swallowing Tongue Pressure Strengthening Exercises in Older Adults: Longitudinal Observational Study. JMIR Mhealth Uhealth (2020) 8(10):e22080. 10.2196/22080 33012704 PMC7600016

[227] Hong E Jakacic AN Sahoo A Breyman E Ukegbu G Tabacof L Use of Fitbit Technology Does Not Impact Health Biometrics in a Community of Older Adults. Telemed e-Health (2021) 27(4):409–13. 10.1089/tmj.2020.0060 32522097

[228] Rocha LA Almeida SS. Saphiraweb: An Open-Source Cloud Platform for E-Health Analysis. Instrum Sci Technol (2020) 48(6):583–600. 10.1080/10739149.2020.1761380

[229] Cid A Sotelo R Leguisamo M Ramírez-Michelena M. Tablets for Deeply Disadvantaged Older Adults: Challenges in Long-Term Care Facilities. Int J Hum Comput Stud (2020) 144. 10.1016/j.ijhcs.2020.102504

[230] Zulfiqar AA Massimbo DND Hajjam M Geny B Talha S Hajjam J Results of the Second Phase of the Ger-E-Tec Experiment Concerning the Telemonitoring of Elderly Patients Affected by Covid-19 Disease to Detect the Exacerbation of Geriatric Syndromes. J Pers Med (2021) 11(11):1117. 10.3390/jpm11111117 34834469 PMC8621367

[231] Zulfiqar AA Vaudelle O Hajjam M Geny B Talha S Letourneau D Results of the “GER-e-TEC” Experiment Involving the Use of an Automated Platform to Detect the Exacerbation of Geriatric Syndromes. J Clin Med (2020) 9(12):3836–17. 10.3390/jcm9123836 33256080 PMC7761279

[232] Tabbara M Özgüler OS Exadaktylos AK. New Freedom Through Medical Devices Based on the Global System for Mobile Communications: A Prospective Survey of 620 Users of the Swiss Limmex Emergency Wristwatch—An Original Study From Switzerland. Emerg Med Int (2013) 2013:563731–4. 10.1155/2013/563731 24062951 PMC3767057

[233] Van ’t Klooster JWJR Van Der Heide LA Van ’t Klooster JW Van Bruinessen I Boessen A Van Der Heide L Introducing Personalised Self-Management to Support Physical Activity in Elderly: A Case Study. In: International Conference E-Health (2017). Available from: https://www.researchgate.net/publication/319137123 (Accessed July 18, 2023).

[234] Dorrian C Mcgibbon F. Lochaber Telemedicine Clinic-A New Approach Managing Dementia in Care Homes Conference Abstract Lochaber Telemedicine Clinic-A New Approach Managing Dementia in Care Homes. International Congress on Telehealth and Telecare (2013). Available from: https://www.researchgate.net/publication/296705067 (Accessed July 18, 2023). 10.5334/ijic.1412

[235] Pearlman-Avnion S Goldschmidt Y Shamis N. Impacts of Urbanization and ICT Use on Loneliness Among the Elderly in Israel. Educ Urban Soc (2020) 52(6):962–83. 10.1177/0013124519891998

[236] Saccavini C Mancin S. Integration of Telehealth and Telecare Service in the Veneto Region. Int J Integr Care (2013) 13. 10.5334/ijic.1426

[237] Stellato K Radini D Pellizzari M Pordenon M Pletti L Humar F Integrating Care in Complex Cardiac Care: The Tech Touch. Int J Integr Care (2016) 16(6):383. 10.5334/ijic.2931

[238] Gris S Sampognaro E Forestiero A Saccavini C Mancin S. Improving Integrated Care for Frail Elderly Patients Throught Ict – The Veneto Region Experience. Int J Integr Care (2017) 17(5):520. 10.5334/ijic.3840

[239] Yeung A Johnson DP Trinh NH Weng WCC Kvedar J Fava M. Feasibility and Effectiveness of Telepsychiatry Services for Chinese Immigrants in a Nursing Home. Telemed e-Health. (2009) 15(4):336–41. 10.1089/tmj.2008.0138 19441951

[240] Salerno D. Development of E-Health Services and Integrated/Coordinated Cares. Int J Integr Care (2015) 15. 10.5334/ijic.2144

[241] Robič M Pavlič DR. COVID-19 and Care for the Elderly in Long-Term Care Facilities: The Role Information Communication Technology. Acta Med Academica. Acad Sci Arts Bosnia Herzegovina (2021) 50:414–22. 10.5644/ama2006-124.363 35164520

[242] Casey J. Carers Assistive Technology Service. In: International Digital Health and Care Congress. London (2014). Available from: http://www.ijic.org/ (Accessed July 18, 2023).

[243] Slone S Marshall K Grace P Wall G. Avoidance of Unnecessary Hospital Admissions Through the Implementation of a Digital Health Programme. Int J Integr Care (2018) 18(s2):219. 10.5334/ijic.s2219

[244] Saracchini R Catalina C Bordoni L. A Mobile Augmented Reality Assistive Technology for the Elderly. Comunicar (2015) 23(45):65–73. 10.3916/C45-2015-07

[245] Vázquez-De Sebastián J Ciudin A Castellano-Tejedor C. Analysis of Effectiveness and Psychological Techniques Implemented in Mhealth Solutions for Middle-Aged and Elderly Adults With Type 2 Diabetes: A Narrative Review of the Literature. J Clin Med (2021) 10:2701. 10.3390/jcm10122701 34207402 PMC8235068

[246] Scherr D Kastner P Kollmann A Hallas A Auer J Krappinger H Effect of Home-Based Telemonitoring Using Mobile Phone Technology on the Outcome of Heart Failure Patients After an Episode of Acute Decompensation: Randomized Controlled Trial. J Med Internet Res (2009) 11(3):e34. 10.2196/jmir.1252 19687005 PMC2762855

[247] Magnusson L Hanson E Borg M. A literature review study of Information and Communication Technology as a support for frail older people living at home and their family carers. Technology and Disability (2004) 16(4):223–235. 10.3233/TAD-2004-16404

[248] Egede LE Acierno R Knapp RG Walker RJ Payne EH Christopher Frueh B. Psychotherapy for Depression in Older Veterans via Telemedicine: Effect on Quality of Life, Satisfaction, Treatment Credibility, and Service Delivery Perception. J Clin Psychiatry (2016) 77(12):1704–11. 10.4088/JCP.16m10951 27835713

[249] Antonicelli R Mazzanti I Abbatecola AM Parati G. Impact of Home Patient Telemonitoring on Use of β-Blockers in Congestive Heart Failure. Drugs Aging (2010) 27(10):801–5. 10.2165/11538210-000000000-00000 20883060

[250] Trief PM Izquierdo R Eimicke JP Teresi JA Goland R Palmas W Adherence to Diabetes Self Care for White, African-American and Hispanic American Telemedicine Participants: 5 Year Results From the IDEATel Project. Ethn Health (2013) 18(1):83–96. 10.1080/13557858.2012.700915 22762449

[251] Carretero S Centeno C Stewart J. Telecare and Telehealth for Informal Carers: A Research in 12 Member States on Their Benefits and Policy Role for the Success. Int J Integr Care (2013) 13(7). 10.5334/ijic.1374

[252] Poon P Hui E Dai D Kwok T Woo J. Cognitive Intervention for Community-Dwelling Older Persons With Memory Problems: Telemedicine Versus Face-To-Face Treatment. Int J Geriatr Psychiatry (2005) 20(3):285–6. 10.1002/gps.1282 15717335

[253] West SP Lagua C Trief PM Izquierdo R Weinstock RS. Goal Setting Using Telemedicine in Rural Underserved Older Adults With Diabetes: Experiences From the Informatics for Diabetes Education and Telemedicine Project. Available from: www.liebertpub.com (Accessed July 19, 2023). 10.1089/tmj.2009.0136 20507198

[254] Zaccarelli C Benati G Boschi F Barban F Annicchiarico R Lymperopoulou O. Computer-Based Cognitive Training for Dementia. Results From a Randomized Controlled Trial on MCI, Mild AD and Healthy Ageing. In: Journal of Alzheimer’s Disease. IOS Press (2016). p. S56. 10.3233/JAD-169001

[255] Comín-Colet J Enjuanes C Verdú-Rotellar JM Linas A Ruiz-Rodriguez P González-Robledo G Impact on Clinical Events and Healthcare Costs of Adding Telemedicine to Multidisciplinary Disease Management Programmes for Heart Failure: Results of a Randomized Controlled Trial. J Telemed Telecare (2016) 22(5):282–95. 10.1177/1357633X15600583 26350543

[256] Jenkins R McSweeney M. Assessing Elderly Patients With Congestive Heart Failure via in-Home Interactive Telecommunication. J Gerontol Nurs (2001) 27:21–7. 10.3928/0098-9134-20010101-09 11915093

[257] Gathercole R Bradley R Harper E Davies L Pank L Lam N Assistive Technology and Telecare to Maintain Independent Living at Home for People With Dementia: The ATTILA RCT. Health Technol Assess (Rockv) (2021) 25(19):1–156. 10.3310/hta25190 33755548 PMC8020444

[258] Pedone C Chiurco D Scarlata S Incalzi RA. Efficacy of Multiparametric Telemonitoring on Respiratory Outcomes in Elderly People With COPD: A Randomized Controlled Trial. BMC Health Serv Res (2013) 13:82. 10.1186/1472-6963-13-82 23497109 PMC3680224

[259] Antonicelli R Testarmata P Spazzafumo L Gagliardi C Bilo G Valentini M Impact of Telemonitoring at Home on the Management of Elderly Patients With Congestive Heart Failure. J Telemed Telecare (2008) 14(6):300–5. 10.1258/jtt.2008.071213 18776075

[260] Sabatier R Coutance G Belin A Biannic C Loiselet P Pradere G Three Months Educational Remote Telemonitoring in Elderly Patients With Heart Failure Reduces Hospitalizations for Acute Heart Failure at One Year: A Randomized Trial. P666. In: European Journal of Heart Failure. Wiley (2013).

[261] Mavandadi S Benson A DiFilippo S Streim JE Oslin D. A Telephone-Based Program to Provide Symptom Monitoring Alone vs Symptom Monitoring Plus Care Management for Late-Life Depression and Anxiety a Randomized Clinical Trial. JAMA Psychiatry (2015) 72(12):1211–8. 10.1001/jamapsychiatry.2015.2157 26558530

[262] Zeghari R Guerchouche R Tran Duc M Bremond F Lemoine MP Bultingaire V Pilot Study to Assess the Feasibility of a Mobile Unit for Remote Cognitive Screening of Isolated Elderly in Rural Areas. Int J Environ Res Public Health (2021) 18(11):6108. 10.3390/ijerph18116108 34198917 PMC8201036

[263] Sharma Y Thompson CH Kaambwa B Shahi R Hakendorf P Miller M. Investigation of the Benefits of Early Malnutrition Screening With Telehealth Follow up in Elderly Acute Medical Admissions. QJM: Int J Med (2017) 110(10):639–47. 10.1093/qjmed/hcx095 28472530

[264] Trief PM Teresi JA Izquierdo R Morin PC Goland R Field L Psychosocial Outcomes of Telemedicine Case Management for Elderly Patients With Diabetes: The Randomized IDEATel Trial. Diabetes Care (2007) 30:1266–8. 10.2337/dc06-2476 17325261

[265] Mandal A Ng N Mok V Lantz M Sinai M Israel B “Put It on Mi Tab”: Assessing the Cost of Geriatric “Regulars” in the Psychiatric Emergency Department. In: AAGP Annual Meeting (2019).

[266] Noel HC Vogel DC Erdos JJ Cornwall D Levin F. Home Telehealth Reduces Healthcare Costs. Telemed J E Health (2004) 10:170–83. 10.1089/tmj.2004.10.170 15319047

[267] Hirani SP Beynon M Cartwright M Rixon L Doll H Henderson C The Effect of Telecare on the Quality of Life and Psychological Well-Being of Elderly Recipients of Social Care over a 12-Month Period: The Whole Systems Demonstrator Cluster Randomised Trial. Age Ageing (2014) 43(3):334–41. 10.1093/ageing/aft185 24333802

[268] Gellis ZD Kenaley BL Have TT. Integrated Telehealth Care for Chronic Illness and Depression in Geriatric Home Care Patients: The Integrated Telehealth Education and Activation of Mood (I-TEAM) Study. J Am Geriatr Soc (2014) 62(5):889–95. 10.1111/jgs.12776 24655228

[269] Sorknaes AD Bech M Madsen H Titlestad IL Hounsgaard L Hansen-Nord M The Effect of Real-Time Teleconsultations Between Hospital-Based Nurses and Patients With Severe COPD Discharged After an Exacerbation. J Telemed Telecare (2013) 19(8):466–74. 10.1177/1357633X13512067 24227799

[270] Lin FC Chien HY Chen SH Kao YC Cheng PW Te WC. Voice Therapy for Benign Voice Disorders in the Elderly: A Randomized Controlled Trial Comparing Telepractice and Conventional Face-To-Face Therapy. J Speech, Lang Hearing Res (2020) 63(7):2132–40. 10.1044/2020_JSLHR-19-00364 32579859

[271] Cassel S. A Comparison of Traditional Face-To-Face and Tele-Dysphagia Instructional Methods in Geriatric TBI and CVA Populations. Arch Phys Med Rehabil (2016) 97(10):e16–7. 10.1016/j.apmr.2016.08.046

[272] Titov N Dear BF Ali S Zou JB Lorian CN Johnston L Clinical and Cost-Effectiveness of Therapist-Guided Internet-Delivered Cognitive Behavior Therapy for Older Adults With Symptoms of Depression: A Randomized Controlled Trial. Behav Ther (2015) 46:193–205. 10.1016/j.beth.2014.09.008 25645168

[273] Pedone C Rossi FF Cecere A Costanzo L Antonelli Incalzi R. Efficacy of a Physician-Led Multiparametric Telemonitoring System in Very Old Adults With Heart Failure. J Am Geriatr Soc (2015) 63(6):1175–80. 10.1111/jgs.13432 26031737

[274] Choi NG Hegel MT Nathan Marti C Lynn Marinucci M Sirrianni L Bruce ML. Telehealth Problem-Solving Therapy for Depressed Low-Income Homebound Older Adults. Am J Geriatr Psychiatry (2012) 1:1. 10.1097/jgp.0b013e318266b356 23567376 PMC3519946

[275] Lin KH Chen CH Chen YY Huang WT Lai JS Yu SM Bidirectional and Multi-User Telerehabilitation System: Clinical Effect on Balance, Functional Activity, and Satisfaction in Patients With Chronic Stroke Living in Long-Term Care Facilities. Sensors (Switzerland) (2014) 14(7):12451–66. 10.3390/s140712451 25019632 PMC4168417

[276] Baker K LaValley MP Brown C Felson DT Ledingham A Keysor JJ. Efficacy of Computer-Based Telephone Counseling on Long-Term Adherence to Strength Training in Elderly Patients With Knee Osteoarthritis: A Randomized Trial. Arthritis Care Res (Hoboken) (2020) 72(7):982–90. 10.1002/acr.23921 31074576 PMC10521167

[277] Sparrow D Gottlieb DJ Demolles D Fielding RA. Increases in Muscle Strength and Balance Using a Resistance Training Program Administered via a Telecommunications System in Older Adults. Journals Gerontol - Ser A Biol Sci Med Sci (2011) 66 A(11):1251–7. 10.1093/gerona/glr138 21852283 PMC3304299

[278] Jakobsen AS Laursen LC Rydahl-Hansen S Østergaard B Gerds TA Emme C Home-Based Telehealth Hospitalization for Exacerbation of Chronic Obstructive Pulmonary Disease: Findings From “The Virtual Hospital Trial”. Telemed e-Health (2015) 21(5):364–73. 10.1089/tmj.2014.0098 25654366 PMC4432494

[279] Olivari Z Giacomelli S Gubian L Mancin S Visentin E Di Francesco V The Effectiveness of Remote Monitoring of Elderly Patients After Hospitalisation for Heart Failure: The Renewing Health European Project. Int J Cardiol (2018) 257:137–42. 10.1016/j.ijcard.2017.10.099 29506685

[280] Tieu C Chaudhry R Schroeder DR Bock FA Hanson GJ Tung EE. Utilization of Patient Electronic Messaging to Promote Advance Care Planning in the Primary Care Setting. Am J Hosp Palliat Med (2017) 34(7):665–70. 10.1177/1049909116650237 27188759

[281] Valdivieso B García-Sempere A Sanfélix-Gimeno G Faubel R Librero J Soriano E The Effect of Telehealth, Telephone Support or Usual Care on Quality of Life, Mortality and Healthcare Utilization in Elderly High-Risk Patients With Multiple Chronic Conditions. A Prospective Study. Med Clin (Barc) (2018) 151(8):308–14. 10.1016/j.medcli.2018.03.013 29705155

[282] Barnason S Zimmerman L Nieveen J Hertzog M. Impact of a Telehealth Intervention to Augment Home Health Care on Functional and Recovery Outcomes of Elderly Patients Undergoing Coronary Artery Bypass Grafting. Heart Lung J Acute Crit Care (2006) 35(4):225–33. 10.1016/j.hrtlng.2005.10.003 16863894

[283] Pandey A Choudhry N. An M-Health Application of Daily Text Messages to Address Forgetfulness as Contributor to Non-Adherence to Medications Post-Myocardial Infarction. In: Welcome to the Future: Technological Changes to Cardiovascular Health and Health Care (2015).

[284] Guo Y Lip GYH. Improving Outcomes With Integrated Care of Patients With Atrial Fibrillation and Multimorbidity Using Mobile Health Technology: A Report From the mAFA II Trial. Available from: https://academic.oup.com/eurheartj/article/42/Supplement_1/ehab724.3114/6391995 (Accessed July 5, 2023).

[285] Zimmerman L Barnason S Nieveen J Schmaderer M. Symptom Management Intervention in Elderly Coronary Artery Bypass Graft Patients. 14740578

[286] Shany T Hession M Pryce D Galang R Roberts M Lovell N Home Telecare Study for Patients With Chronic Lung Disease in the Sydney West Area Health Service. In: Studies in Health Technology and Informatics. IOS Press (2010). p. 139–48. 10.3233/978-1-60750-659-1-139 21191167

[287] Fottrell E Ahmed N Morrison J Kuddus A Shaha SK King C Community Groups or Mobile Phone Messaging to Prevent and Control Type 2 Diabetes and Intermediate Hyperglycaemia in Bangladesh (DMagic): A Cluster-Randomised Controlled Trial. Lancet Diabetes Endocrinol (2019) 7(3):200–12. 10.1016/S2213-8587(19)30001-4 30733182 PMC6381080

[288] De Kluiver EP Van Der Velde AE Meindersma EP Prins LF Wilhelm M Iliou MC A European Randomised Controlled Trial for M-Health Guided Cardiac Rehabilitation in the Elderly; Results of the EU-CaRE RCT Study. In: ESC Congress 2019 Together with World Congress of Cardiology (2019). Available from: https://academic.oup.com/eurheartj/article/40/Supplement_1/ehz748.0674/5597461 (Accessed July 5, 2023).

[289] Yu F Mathiason MA Johnson K Gaugler JE Klassen D. Memory Matters in Dementia: Efficacy of a Mobile Reminiscing Therapy App. Alzheimer’s Demen Translational Res Clin Interventions (2019) 5:644–51. 10.1016/j.trci.2019.09.002 31720365 PMC6838539

[290] Gschwind YJ Eichberg S Ejupi A de Rosario H Kroll M Marston HR ICT-Based System to Predict and Prevent Falls (iStoppFalls): Results From an International Multicenter Randomized Controlled Trial. Eur Rev Aging Phys Activity (2015) 12(1):10. 10.1186/s11556-015-0155-6 26865874 PMC4748323

[291] Núñez-Naveira L Alonso-Búa B De Labra C Gregersen R Maibom K Mojs E UnderstAID, an ICT Platform to Help Informal Caregivers of People With Dementia: A Pilot Randomized Controlled Study. Biomed Res Int (2016) 2016:5726465. 10.1155/2016/5726465 28116300 PMC5225394

[292] Brath H Morak J Kästenbauer T Modre-Osprian R Strohner-Kästenbauer H Schwarz M Mobile Health (mHealth) Based Medication Adherence Measurement - A Pilot Trial Using Electronic Blisters in Diabetes Patients. Br J Clin Pharmacol (2013) 76(S1):47–55. 10.1111/bcp.12184 24007452 PMC3781679

[293] Chan MHM Keung DTF Lui SYT Cheung RTH. A Validation Study of a Smartphone Application for Functional Mobility Assessment of the Elderly. Hong Kong Physiother J (2016) 35:1–4. 10.1016/j.hkpj.2015.11.001 30931027 PMC6385142

[294] Wade M Desai A Spettell CM Snyder AD McGowan-Stackewicz V Kummer PJ Telemonitoring With Case Management for Seniors With Heart Failure. Am J Manag Care (2011) 17(3):e71–e79. 21504262

[295] Shapira S Yeshua-Katz D Cohn-Schwartz E Aharonson-Daniel L Sarid O Clarfield AM. A Pilot Randomized Controlled Trial of a Group Intervention via Zoom to Relieve Loneliness and Depressive Symptoms Among Older Persons During the COVID-19 Outbreak. Internet Interv (2021) 24. 10.1016/j.invent.2021.100368 33527072 PMC7839498

[296] Chan JYC Chan TK Wong MPF Cheung RSM Yiu KKL Tsoi KKF. Effects of Virtual Reality on Moods in Community Older Adults. A Multicenter Randomized Controlled Trial. Int J Geriatr Psychiatry (2020) 35(8):926–33. 10.1002/gps.5314 32346896

[297] Pérez-Rodríguez R Guevara-Guevara T Moreno-Sánchez PA Villalba-Mora E Valdés-Aragonés M Oviedo-Briones M Monitoring and Intervention Technologies to Manage Diabetic Older Persons: The CAPACITY Case—A Pilot Study. Front Endocrinol (Lausanne) (2020) 13:11. 10.3389/fendo.2020.00300 32528409 PMC7247856

[298] Guo Y Chen Y Lane DA Liu L Wang Y Lip GYH. Mobile Health Technology for Atrial Fibrillation Management Integrating Decision Support, Education, and Patient Involvement: mAF App Trial. Am J Med (2017) 130(12):1388–96. 10.1016/j.amjmed.2017.07.003 28847546

[299] Li X Li T Chen J Xie Y An X Lv Y A Wechat-Based Self-Management Intervention for Community Middle-Aged and Elderly Adults With Hypertension in Guangzhou, China: A Cluster-Randomized Controlled Trial. Int J Environ Res Public Health (2019) 16(21):4058. 10.3390/ijerph16214058 31652688 PMC6862068

[300] Finkelstein SM Speedie SM Zhou X Potthoff S Ratner ER. Perception, Satisfaction and Utilization of the VALUE Home Telehealth Service. J Telemed Telecare (2011) 17(6):288–92. 10.1258/jtt.2011.100712 21844178

[301] Guo Y Lane DA Chen Y Lip GYH mAF-App II Trial investigators. Regular Bleeding Risk Assessment Associated With Reduction in Bleeding Outcomes: The mAFA-II Randomized Trial. Am J Med (2020) 133(10):1195–202.e2. 10.1016/j.amjmed.2020.03.019 32289310

[302] Hermes S Lurz M Böhm M Krcmar H. Evaluating the Usability and Usefulness of a Mobile Application for Training Visual Mnemonic Techniques in Participants With Subjective Cognitive Decline: An Exploratory Pilot Study. In: Procedia Computer Science. Elsevier B.V. (2019). p. 439–44.

[303] Mira JJ Navarro I Botella F Borrás F Nuño-Solinís R Orozco D A Spanish Pillbox App for Elderly Patients Taking Multiple Medications: Randomized Controlled Trial. J Med Internet Res (2014) 16(4):e99. 10.2196/jmir.3269 24705022 PMC4004137

[304] Mertens A Brandl C Miron-Shatz T Schlick C Neumann T Kribben A A Mobile Application Improves Therapy-Adherence Rates in Elderly Patients Undergoing Rehabilitation A Crossover Design Study Comparing Documentation via iPad With Paper-Based Control. Medicine (United States) (2016) 95(36):e4446. 10.1097/MD.0000000000004446 27603339 PMC5023861

[305] Robert P Manera V Derreumaux A Montesino MFY Leone E Fabre R Efficacy of a Web App for Cognitive Training (MEMO) Regarding Cognitive and Behavioral Performance in People With Neurocognitive Disorders: Randomized Controlled Trial. J Med Internet Res (2020) 22(3):e17167. 10.2196/17167 32159519 PMC7097721

[306] Ki JY Jo SR Cho KS Park JE Cho JW Jang JH. Effect of Oral Health Education Using a Mobile App (Ohema) on the Oral Health and Swallowing-Related Quality of Life in Community-Based Integrated Care of the Elderly: A Randomized Clinical Trial. Int J Environ Res Public Health (2021) 18(21):11679. 10.3390/ijerph182111679 34770193 PMC8582748

[307] Snoek JA Prescott EI Van Der Velde AE Eijsvogels TMH Mikkelsen N Prins LF Effectiveness of Home-Based Mobile Guided Cardiac Rehabilitation as Alternative Strategy for Nonparticipation in Clinic-Based Cardiac Rehabilitation Among Elderly Patients in Europe: A Randomized Clinical Trial. JAMA Cardiol (2021) 6(4):463–8. 10.1001/jamacardio.2020.5218 33112363 PMC7593879

[308] Yu J Jeon H Song C Han W. Speech Perception Enhancement in Elderly Hearing Aid Users Using an Auditory Training Program for Mobile Devices. Geriatr Gerontol Int (2017) 17(1):61–8. 10.1111/ggi.12678 26628069

[309] Tchalla AE Lachal F Cardinaud N Saulnier I Rialle V Preux PM Preventing and Managing Indoor Falls With Home-Based Technologies in Mild and Moderate Alzheimer’s Disease Patients: Pilot Study in a Community Dwelling. Dement Geriatr Cogn Disord (2013) 36(3–4):251–61. 10.1159/000351863 23949277

[310] Berkman P Heinik J Rosenthal M Burke M. Supportive Telephone Outreach as an Interventional Strategy for Elderly Patients in a Period of Crisis. Soc Work Health Care (1999) 28(4):63–76. 10.1300/J010v28n04_05 10425672

[311] Tchalla AE Lachal F Cardinaud N Saulnier I Bhalla D Roquejoffre A Efficacy of Simple Home-Based Technologies Combined With a Monitoring Assistive Center in Decreasing Falls in a Frail Elderly Population (Results of the Esoppe Study). Arch Gerontol Geriatr (2012) 55(3):683–9. 10.1016/j.archger.2012.05.011 22743136

[312] Upatising B Hanson GJ Kim YL Cha SS Yih Y Takahashi PY. Effects of Home Telemonitoring on Transitions Between Frailty States and Death for Older Adults: A Randomized Controlled Trial. Int J Gen Med (2013) 6:145–51. 10.2147/IJGM.S40576 23525664 PMC3603330

[313] Read Paul L Salmon C Sinnarajah A Spice R. Web-Based Videoconferencing for Rural Palliative Care Consultation With Elderly Patients at Home. Support Care Cancer (2019) 27(9):3321–30. 10.1007/s00520-018-4580-8 30613908

[314] Chang R Low H McDonald A Park G Song X. Web-Based Software Applications for Frailty Assessment in Older Adults: A Scoping Review of Current Status With Insights Into Future Development. BMC Geriatr (2021) 21(1):723. 10.1186/s12877-021-02660-6 34922466 PMC8683817

[315] Kim HH Lee SH Cho NB You H Choi T Kim J. User-Dependent Usability and Feasibility of a Swallowing Training Mhealth App for Older Adults: Mixed Methods Pilot Study. JMIR Mhealth Uhealth (2020) 8(7):e19585. 10.2196/19585 32663161 PMC7418014

[316] Chan WM Woo J Hui E Hjelm NM. The Role of Telenursing in the Provision of Geriatric Outreach Services to Residential Homes in Hong Kong. J Telemed Telecare (2001) 7:38–46. 10.1258/1357633011936129 11265937

[317] Richard S Lavandier K Zioueche Y Pelletier S Vezain A Ducrocq X. Use of Telemedicine to Manage Severe Ischaemic Strokes in a Rural Area With an Elderly Population. Neurol Sci (2014) 35(5):683–5. 10.1007/s10072-013-1577-4 24277200

[318] Güttler J Georgoulas C Linner T Bock T. Towards a Future Robotic Home Environment: A Survey. Gerontology (2015) 61:268–80. 10.1159/000363698 25341658

[319] Ohligs M Stocklassa S Rossaint R Czaplik M Follmann A. Employment of Telemedicine in Nursing Homes: Clinical Requirement Analysis, System Development and First Test Results. Clin Interv Aging (2020) 15:1427–37. 10.2147/CIA.S260098 32884251 PMC7443448

[320] De Luca R Bramanti A De Cola MC Trifiletti A Tomasello P Torrisi M Tele-Health-Care in the Elderly Living in Nursing Home: The First Sicilian Multimodal Approach. Aging Clin Exp Res (2016) 28(4):753–9. 10.1007/s40520-015-0463-8 26420423

[321] Matsumoto Y Kizaki H Ikeda Y Nakamura S Kina S Nagai T Telepharmacy in Mountainous Depopulated Areas of Japan: An Exploratory Interview Study of Patients’ Perspectives. Drug Discov Ther (2021) 15(6):337–40. 10.5582/ddt.2021.01102 34980762

[322] Persson HL Lyth J Lind L. The Health Diary Telemonitoring and Hospital-Based Home Care Improve Quality of Life Among Elderly Multimorbid Copd and Chronic Heart Failure Subjects. Int J COPD (2020) 15:527–41. 10.2147/COPD.S236192 32210547 PMC7069558

[323] Goodman-Casanova JM Dura-Perez E Guzman-Parra J Cuesta-Vargas A Mayoral-Cleries F. Telehealth Home Support During COVID-19 Confinement for Community-Dwelling Older Adults With Mild Cognitive Impairment or Mild Dementia: Survey Study. J Med Internet Res (2020) 22:e19434. 10.2196/19434 32401215 PMC7247465

[324] Hao JF Cui HM Han JM Bai JX Song X Cao N. Tele-ICU: The Way Forward in Geriatric Care? In: Aging Clinical and Experimental Research. Springer International Publishing (2014). 26:575–82. 24803284 10.1007/s40520-014-0217-z

[325] Choo CC Kuek JHL Burton AAD. Smartphone Applications for Mindfulness Interventions With Suicidality in Asian Older Adults: A Literature Review. Int J Environ Res Public Health MDPI AG (2018) 15:2810. 10.3390/ijerph15122810 30544738 PMC6313610

[326] Sadek I Mohktari M. Nonintrusive Remote Monitoring of Sleep in Home-Based Situation. J Med Syst (2018) 42(4):64. 10.1007/s10916-018-0917-6 29497864

[327] Aanesen M Lotherington AT Olsen F. Smarter Elder Care? A Cost-Effectiveness Analysis of Implementing Technology in Elder Care. Health Inform J (2011) 17(3):161–72. 10.1177/1460458211409716 21937460

[328] Barnes N Senior SW Tom R Researcher M Researcher JN Reeves A. Liverpool Telecare Pilot: Case Studies Mark Buckland Senior Researcher.

[329] Bartels SJ DiMilia PR Fortuna KL Naslund JA. Integrated Care for Older Adults With Serious Mental Illness and Medical Comorbidity: Evidence-Based Models and Future Research Directions. Psychiatr Clin North America (2018) 41:153–64. 10.1016/j.psc.2017.10.012 29412843 PMC5806142

[330] Li F Harmer P Voit J Chou LS. Implementing an Online Virtual Falls Prevention Intervention During a Public Health Pandemic for Older Adults With Mild Cognitive Impairment: A Feasibility Trial. Clin Interv Aging (2021) 16:973–83. 10.2147/CIA.S306431 34079243 PMC8164667

[331] de Batlle J Massip M Vargiu E Nadal N Fuentes A Bravo MO Implementing Mobile Health-Enabled Integrated Care for Complex Chronic Patients: Intervention Effectiveness and Cost-Effectiveness Study. JMIR Mhealth Uhealth (2021) 9(1):e22135. 10.2196/22135 33443486 PMC7843204

[332] Matz O Villa L Lecce C Olaciregui Dague K Haeger A Bollheimer LC Implementation of a Telemedicine Geriatric Co-Evaluation in the Emergency Department: A Prospective Pilot Study. Swiss Med Wkly (2021) 151:w20500. 10.4414/smw.2021.20500 34000061

[333] Nogués X Sánchez-Martinez F Castells X Díez-Pérez A Sabaté RA Petit I Hospital-at-Home Expands Hospital Capacity During COVID-19 Pandemic. J Am Med Dir Assoc (2021) 22(5):939–42. 10.1016/j.jamda.2021.01.077 33639115 PMC7847393

[334] Rubegni P Nami N Cevenini G Poggiali S Hofmann-Wellenhof R Massone C Geriatric Teledermatology: Store-And-Forward vs. Face-To-Face Examination. J Eur Acad Dermatol Venereol (2011) 25(11):1334–9. 10.1111/j.1468-3083.2011.03986.x 21349115

[335] Kikuchi A Taniguchi T Nakamoto K Sera F Ohtani T Yamada T Feasibility of Home-Based Cardiac Rehabilitation Using an Integrated Telerehabilitation Platform in Elderly Patients With Heart Failure: A Pilot Study. J Cardiol (2021) 78(1):66–71. 10.1016/j.jjcc.2021.01.010 33579602

[336] Hoffmann W van den Berg N Thyrian JR Fiss T. Frequency and Determinants of Potential Drug-Drug Interactions in an Elderly Population Receiving Regular Home Visits by GPs - Results of the Home Medication Review in the AGnES-Studies. Pharmacoepidemiol Drug Saf (2011) 20(12):1311–8. 10.1002/pds.2224 21919114

[337] Jelcic N Agostini M Meneghello F Bussè C Parise S Galano A Feasibility and Efficacy of Cognitive Telerehabilitation in Early Alzheimer’s Disease: A Pilot Study. Clin Interv Aging (2014) 9:1605–11. 10.2147/CIA.S68145 25284993 PMC4181448

[338] Skjæret N Nawaz A Morat T Schoene D Helbostad JL Vereijken B. Exercise and Rehabilitation Delivered Through Exergames in Older Adults: An Integrative Review of Technologies, Safety and Efficacy. Int J Med Inform (2016) 85:1–16. 10.1016/j.ijmedinf.2015.10.008 26559887

[339] Choi H Kim J. Effectiveness of Telemedicine: Videoconferencing for Low-Income Elderly With Hypertension. Telemed e-Health (2014) 20(12):1156–64. 10.1089/tmj.2014.0031 25469880 PMC4270109

[340] Scherrenberg M Zeymer U Schneider S Van der Velde AE Wilhelm M Van’t Hof AWJ EU-CaRE Study: Could Exercise-Based Cardiac Telerehabilitation Also Be Cost-Effective in Elderly? Int J Cardiol (2021) 340:1–6. 10.1016/j.ijcard.2021.08.024 34419529

[341] Persson HL Lyth J Wiréhn AB Lind L. Elderly Patients With COPD Require More Health Care Than Elderly Heart Failure Patients Do in a Hospital-Based Home Care Setting. Int J COPD (2019) 14:1569–81. 10.2147/COPD.S207621 31406459 PMC6642647

[342] Koceska N Koceski S Zobel PB Trajkovik V Garcia N. A Telemedicine Robot System for Assisted and Independent Living. Sensors (Switzerland) (2019) 19(4):834. 10.3390/s19040834 30781647 PMC6412532

[343] García‐villamil G Neira‐álvarez M Huertas‐hoyas E Ramón‐jiménez A Rodríguez‐sánchez C. A Pilot Study to Validate a Wearable Inertial Sensor for Gait Assessment in Older Adults With Falls. Sensors (2021) 21(13):4334. 10.3390/s21134334 34202786 PMC8272102

[344] Tegeler C Beyer AK Hoppmann F Ludwig V Kessler E-M. Current State of Research on Psychotherapy for Home-Living Vulnerable Older Adults With Depression. Z Gerontologie Geriatrie (2020) 53:721–7. 10.1007/s00391-020-01805-3 33185718 PMC7661801

[345] Kraft M Van Den Berg N Kraft K Schmekel S Gärtner S Krüger J Development of a Telemedical Monitoring Concept for the Care of Malnourished Geriatric Home-Dwelling Patients: A Pilot Study. Maturitas (2012) 72(2):126–31. 10.1016/j.maturitas.2012.02.011 22440535

[346] Abbas M Somme D Le Bouquin Jeannès R. D-SORM: A Digital Solution for Remote Monitoring Based on the Attitude of Wearable Devices. Comput Methods Programs Biomed (2021) 208. 10.1016/j.cmpb.2021.106247 34260971

[347] Li C Neugroschl J Zhu CW Aloysi A Schimming CA Cai D Design Considerations for Mobile Health Applications Targeting Older Adults. J Alzheimer’s Dis (2021) 79(1):1–8. 10.3233/JAD-200485 33216024 PMC8837196

[348] Dang Ãwz S Ma F Nedd N Florez Ãw H Ãw EA Roos Ãwz BA. Care Coordination and Telemedicine Improves Glycaemic Control in Ethnically Diverse Veterans With Diabetes. J Telemed Telecare (2007) 13:263–7. 10.1258/135763307781458958 17697515

[349] Gamito P Oliveira J Morais D Coelho C Santos N Alves C Cognitive Stimulation of Elderly Individuals With Instrumental Virtual Reality-Based Activities of Daily Life: Pre-Post Treatment Study. Cyberpsychol Behav Soc Netw (2019) 22(1):69–75. 10.1089/cyber.2017.0679 30040477

[350] Brunetti ND De Gennaro L Pellegrino PL Dellegrottaglie G Antonelli G Di Biase M. Atrial Fibrillation With Symptoms Other Than Palpitations: Incremental Diagnostic Sensitivity With At-Home Tele-Cardiology Assessment for Emergency Medical Service. Eur J Prev Cardiol (2012) 19(3):306–13. 10.1177/1741826711406060 21502279

[351] Göransson C Wengström Y Hälleberg-Nyman M Langius-Eklöf A Ziegert K Blomberg K. An App for Supporting Older People Receiving Home Care - Usage, Aspects of Health and Health Literacy: A Quasi-Experimental Study. BMC Med Inform Decis Mak (2020) 20(1):226. 10.1186/s12911-020-01246-3 32933500 PMC7493150

[352] Sugihara T Fujinami T Phaal R Ikawa Y. A Technology Roadmap of Assistive Technologies for Dementia Care in Japan. Dementia (2015) 14(1):80–103. 10.1177/1471301213493798 24339091

[353] Wong YK Hui E Woo J. A Community-Based Exercise Programme for Older With Knee Pain Using Telemedicine. J Telemed Telecare (2005) 11:310–5. 10.1258/1357633054893346 16168168

[354] Moriichi K Fujiya M Ro T Ota T Nishimiya H Kodama M A Novel Telerehabilitation With an Educational Program for Caregivers Using Telelecture Is Feasible for Fall Prevention in Elderly People: A Case Series. Medicine (United States) (2022) 101(6):E27451. 10.1097/MD.0000000000027451 35147084 PMC8830826

[355] Fasilis T Patrikelis P Siatouni A Alexoudi A Veretzioti A Zachou L A Pilot Study and Brief Overview of Rehabilitation via Virtual Environment in Patients Suffering From Dementia. Psychiatriki (2018) 29(1):42–51. 10.22365/jpsych.2018.291.42 29754119

[356] Fallah M Yasini M. A Medication Reminder Mobile App: Does It Work for Different Age Ranges. Stud Health Technol Inform (2017) 235:68–72. 28423757

[357] Audiau S Schmoll L Blanc F. Déplacements et Hospitalisations Évités Grâce aux Téléconsultations pour les Patients Hébergés en Ehpad. Geriatr Psychol Neuropsychiatr Vieil (2021) 19(4):447–58. 10.1684/pnv.2021.0990 34955457

[358] Andrès E Talha S Benyahia A Keller O Hajjam M Moukadem A Expérimentation d’une Plateforme de Détection Automatisée des Situations à Risque de Décompensation Cardiaque (Plateforme E-Care) dans une Unité de Médecine Interne. Revue de Medecine Interne (2016) 37(9):587–93. 10.1016/j.revmed.2016.01.004 26852082

[359] Brondi R Banno F Bendinelli S Castelli C Mancina A Marinoni M Drugs Don’t Work in Patients Who Don’t Take Them: Dr. Drin, the New ICT Paradigm for Chronic Therapies. Stud Health Technol Inform (2013) 191:100–4. 23792852

[360] Schofield RS Kline SE Schmalfuss CM Carver HM Aranda JM Pauly DF Early Outcomes of a Care Coordination-Enhanced Telehome Care Program for Elderly Veterans With Chronic Heart Failure. Telemed J E Health (2005) 11:20–7. 10.1089/tmj.2005.11.20 15785217

[361] Corcoran H Hui E Woo J. The Acceptability of Telemedicine for Podiatric Intervention in a Residential Home for the Elderly. 10.1258/135763303767149942 12877776

[362] Vestal L Smith-Olinde L Hicks G Hutton T Hart J Jr. Efficacy of Language Assessment in Alzheimer’s Disease: Comparing In-Person Examination and Telemedicine. Clin Interv Aging (2006) 1(4):467–71. 10.2147/ciia.2006.1.4.467 18046923 PMC2699639

[363] Duke C. The Frail Elderly Community– Based Case Management Project. Geriatr Nurs (Minneap) (2005) 26(2):122–7. 10.1016/j.gerinurse.2005.03.003 15824728

[364] Moon Chae Y Heon Lee J Hee Ho S Ja Kim H Hong Jun K Uk Won J. Patient Satisfaction With Telemedicine in Home Health Services for the Elderly. Int J Med Inform (2001) 61:167–73. 10.1016/s1386-5056(01)00139-3 11311671

[365] Hui E Woo J Hjelm M Zhang YT Tsui HT. Clinical Section Telemedicine: A Pilot Study in Nursing Home Residents (2001). Available from: https://www.karger.com/journals/ger (Accessed July 5, 2023). 10.1159/000052778 11287732

[366] Demiris G Shigaki CL Schopp LH. An Evaluation Framework for a Rural Home-Based Telerehabilitation Network. J Med Syst (2005) 29:595–603. 10.1007/s10916-005-6127-z 16235812

[367] Schweickert PA Rutledge CM Cattell-Gordon DC Div MW Solenski NJ Jensen ME Telehealth Stroke Education for Rural Elderly Virginians. 10.1089/tmj.2011.0080 22011051

[368] Chumbler NR Mann WC Wu S Schmid A Kobb R. Veterans Affairs Health Services Research and Development/Rehabilitation Research and Development, Rehabilitation Outcomes Research Center (RORC), North Florida/South Georgia Veterans Health System, 6 Tech Care Coordination Program, North Florida/South Georgia Veterans Health System. Telemedicine Journal e-Health (2004) 10.

[369] Citoni B Figliuzzi I Presta V Volpe M Tocci G. Home Blood Pressure and Telemedicine: A Modern Approach for Managing Hypertension During and After COVID-19 Pandemic. Vol. 29, High Blood Pressure and Cardiovascular Prevention. Adis (2022). 10.1007/s40292-021-00492-4 34855154 PMC8638231

[370] Rehm IC Musić S Carlsson A Scanlan F Silver M Bhar SS. Integrating Web-Based Applications Into Aged Care: Two Case Studies and Discussion. J Clin Psychol Med Settings (2016) 23(3):285–97. 10.1007/s10880-016-9457-8 27073103

[371] Fatyga E Dziȩgielewska-Gȩsiak S Wierzgoń A Stołtny D Muc-Wierzgoń M (2020).The Coronavirus Disease 2019 Pandemic: Telemedicine in Elderly Patients With Type 2 Diabetes Polish Arch Intern Med Medycyna Praktyczna 130. 452–4. 10.20452/pamw.15346 32385978

[372] Porath A Irony A Borobick AS Nasser S Malachi A Fund N Maccabi Proactive Telecare Center for Chronic Conditions - The Care of Frail Elderly Patients. Isr J Health Pol Res (2017) 6(1):68. 10.1186/s13584-017-0192-x 29228992 PMC5724333

[373] Batsis JA Pletcher SN Stahl JE. Telemedicine and Primary Care Obesity Management in Rural Areas - Innovative Approach for Older Adults? Vol. BMC Geriatr (2017) 17:6. 10.1186/s12877-016-0396-x 28056832 PMC5216556

[374] Cardozo L Steinberg J. Telemedicine for Recently Discharged Older Patients. Telemed e-Health (2010) 16:49–55. 10.1089/tmj.2009.0058 20064067

[375] Lee J Kim J Jeong S Choi H Jin M Kim S. A Health Recreation Program for U-Healthcare Clients: Effects on Mental Health. Telemed e-Health (2014) 20(10):930–5. 10.1089/tmj.2013.0323 25290668 PMC4188379

[376] Lattanzio F Abbatecola AM Bevilacqua R Chiatti C Corsonello A Rossi L Advanced Technology Care Innovation for Older People in Italy: Necessity and Opportunity to Promote Health and Wellbeing. J Am Med Dir Assoc (2014) 15(7):457–66. 10.1016/j.jamda.2014.04.003 24836715

[377] Melander-Wikman A Fältholm Y Gard G. Safety vs. Privacy: Elderly Persons’ Experiences of a Mobile Safety Alarm. Health Soc Care Community (2008) 16(4):337–46. 10.1111/j.1365-2524.2007.00743.x 18613909

[378] Konstantinidis EI Billis AS Mouzakidis CA Zilidou VI Antoniou PE Bamidis PD. Design, Implementation, and Wide Pilot Deployment of FitForAll: An Easy to Use Exergaming Platform Improving Physical Fitness and Life Quality of Senior Citizens. IEEE J Biomed Health Inform (2016) 20(1):189–200. 10.1109/JBHI.2014.2378814 26731797

[379] Dang S Dimmick S Kelkar G, Evaluating the Evidence Base for the Use of Home Telehealth Remote Monitoring in Elderly With Heart Failure. Telemed J e-health 15 (2009). p. 783–96. 10.1089/tmj.2009.0028 19831704

[380] Chen YH Lin YH Hung CS Huang CC Yeih DF Chuang PY Clinical Outcome and Cost-Effectiveness of a Synchronous Telehealth Service for Seniors and Nonseniors With Cardiovascular Diseases: Quasi-Experimental Study. J Med Internet Res (2013) 15(4):e87. 10.2196/jmir.2091 23615318 PMC3636317

[381] Maresca G De Cola MC Caliri S De Luca R Manuli A Scarcella I Moving Towards Novel Multidisciplinary Approaches for Improving Elderly Quality of Life: The Emerging Role of Telemedicine in Sicily. J Telemed Telecare (2019) 25(5):318–24. 10.1177/1357633X17753057 29409381

[382] Carolan K Grabowski DC Mehrotra A Hatfield LA. Use of Telemedicine for Emergency Triage in an Independent Senior Living Community: Mixed Methods Study. J Med Internet Res (2020) 22(12):e23014. 10.2196/23014 33331827 PMC7775198

[383] Stutzel MC Filippo MP Sztajnberg A Da Costa RMEM Brites ADS Da Motta LB Multi-Part Quality Evaluation of a Customized Mobile Application for Monitoring Elderly Patients With Functional Loss and Helping Caregivers. BMC Med Inform Decis Mak (2019) 19(1):140. 10.1186/s12911-019-0839-3 31331309 PMC6647294

[384] Willard S Cremers G Man YP Van Rossum E Spreeuwenberg M De Witte L. Development and Testing of an Online Community Care Platform for Frail Older Adults in the Netherlands: A User-Centred Design. BMC Geriatr (2018) 18(1):87. 10.1186/s12877-018-0774-7 29625562 PMC5889547

[385] Torp S Hanson E Hauge S Ulstein I Magnusson L. A Pilot Study of How Information and Communication Technology May Contribute to Health Promotion Among Elderly Spousal Carers in Norway. Health Soc Care Community (2008) 16(1):75–85. 10.1111/j.1365-2524.2007.00725.x 18181817

[386] Marinello R Brunetti E Luppi C Bianca D Tibaldi V Isaia G Telemedicine-Assisted Care of an Older Patient With COVID-19 and Dementia: Bridging the Gap Between Hospital and Home. Aging Clin Exp Res (2021) 33(6):1753–6. 10.1007/s40520-021-01875-2 34003476 PMC8129597

[387] Shah MN Gillespie SM Wood N Wasserman EB Nelson DL Dozier A High-intensity Telemedicine-Enhanced Acute Care for Older Adults: An Innovative Healthcare Delivery Model. J Am Geriatr Soc (2013) 61(11):2000–7. 10.1111/jgs.12523 24164485

[388] Gillespie SM Shah MN Wasserman EB Wood NE Wang H Noyes K Reducing Emergency Department Utilization through Engagement in Telemedicine by Senior Living Communities. Engagement Telemed by Senior Living Communities. Telemed e-Health (2016) 22(6):489–96. 10.1089/tmj.2015.0152 26741194

[389] Gaspar AGM Lapão LV. A Digital Health Service for Elderly People With Balance Disorders and Risk of Falling: A Design Science Approach. Int J Environ Res Public Health (2022) 19(3):1855. 10.3390/ijerph19031855 35162877 PMC8835704

[390] Haimi M Gesser-Edelsburg A. Application and Implementation of Telehealth Services Designed for the Elderly Population During the COVID-19 Pandemic: A Systematic Review. Health Inform J (2022) 28(1):14604582221075561. 10.1177/14604582221075561 35175881 PMC8859483

[391] Shah MN Wasserman EB Wang H Gillespie SM Noyes K Wood NE High-Intensity Telemedicine Decreases Emergency Department Use by Senior Living Community Residents. Telemed e-Health (2016) 22(3):251–8. 10.1089/tmj.2015.0103 26252866

[392] Savolainen L Hanson E Magnusson L Gustavsson T. An Internet-Based Videoconferencing System for Supporting Frail Elderly People and Their Carers. J Telemed Telecare (2008) 14(2):79–82. 10.1258/jtt.2007.070601 18348753

[393] Ngaruiya C Oti S Van De Vijver S Kyobutungi C Free C. Target Women: Equity in Access to mHealth Technology in a Non-Communicable Disease Care Intervention in Kenya. PLoS One (2019) 14(9):e0220834. 10.1371/journal.pone.0220834 31509540 PMC6738613

[394] Gillespie SM Wasserman EB Wood NE Wang H Dozier A Nelson D High-Intensity Telemedicine Reduces Emergency Department Use by Older Adults With Dementia in Senior Living Communities. J Am Med Dir Assoc (2019) 20(8):942–6. 10.1016/j.jamda.2019.03.024 31315813 PMC7213053

[395] Sheeran T Rabinowitz T Lotterman J Reilly CF Brown S Donehower P Feasibility and Impact of Telemonitor-Based Depression Care Management for Geriatric Homecare Patients. Telemed e-Health. (2011) 17(8):620–6. 10.1089/tmj.2011.0011 21780942 PMC3208250

[396] Ramos-Ros R Mateos R Lojo D Conn DK Patterson T. Telepsychogeriatrics: A New Horizon in the Care of Mental Health Problems in the Elderly. Int Psychogeriatrics (2012) 24:1708–24. 10.1017/S1041610212000981 22687259

[397] Marschollek M Mix S Wolf KH Effertz B Haux R Steinhagen-Thiessen E. ICT-Based Health Information Services for Elderly People: Past Experiences, Current Trends, and Future Strategies. Med Inform Internet Med (2007) 32(4):251–61. 10.1080/14639230701692736 18072003

[398] De Cola MC De Luca R Bramanti A Bertè F Bramanti P Calabrò RS. Tele-Health Services for the Elderly: A Novel Southern Italy Family Needs-Oriented Model. J Telemed Telecare (2016) 22(6):356–62. 10.1177/1357633X15604290 26377125

[399] Adami I Foukarakis M Ntoa S Partarakis N Stefanakis N Koutras G Monitoring Health Parameters of Elders to Support Independent Living and Improve Their Quality of Life. Sensors (Switzerland) (2021) 21(2):517–36. 10.3390/s21020517 33450904 PMC7828366

[400] Zulfiqar AA Hajjam A Andrès E. Focus on the Different Projects of Telemedicine Centered on the Elderly in France. Curr Aging Sci (2019) 11(4):202–15. 10.2174/1874609812666190304115426 30836931 PMC6635422

[401] Mulasso A Brustio PR Rainoldi A Zia G Feletti L N’Dja A A Comparison Between an ICT Tool and a Traditional Physical Measure for Frailty Evaluation in Older Adults. BMC Geriatr (2019) 19(1):88. 10.1186/s12877-019-1089-z 30898096 PMC6427849

[402] Smith AC Gray LC. Telemedicine Across the Ages. Med J Aust (2009) 190(1):15–9. 10.5694/j.1326-5377.2009.tb02255.x 19120002

[403] Scalvini S Bernocchi P Zanelli E Comini L Vitacca M, Maugeri Centre for Telehealth and Telecare MCTT. Maugeri Centre for Telehealth and Telecare: A Real-Life Integrated Experience in Chronic Patients. J Telemed Telecare (2018) 24(7):500–7. 10.1177/1357633X17710827 28537509

[404] Gentry MT Lapid MI Rummans TA. Geriatric Telepsychiatry: Systematic Review and Policy Considerations. Am J Geriatr Psychiatry (2019) 27(2):109–27. 10.1016/j.jagp.2018.10.009 30416025

[405] Elie-Lefebvre C Schuster JP Limosin F. Télépsychiatrie: Quelle Place dans les Soins des Sujets Âgés. Geriatr Psychol Neuropsychiatr Vieil (2016) 14(3):325–31. 10.1684/pnv.2016.0625 27651014

[406] Karunanithi M. Monitoring Technology for the Elderly Patient. Expert Rev Med Devices (2007) 4:267–77. 10.1586/17434440.4.2.267 17359231

[407] Lyth J Lind L Persson HL Wiréhn AB. Can a Telemonitoring System Lead to Decreased Hospitalization in Elderly Patients? J Telemed Telecare (2021) 27(1):46–53. 10.1177/1357633X19858178 31291794

[408] Tse MMY Choi KCY Leung RSW. E-Health for Older People: The Use of Technology in Health Promotion. Cyberpsychology Behav (2008) 11(4):475–9. 10.1089/cpb.2007.0151 18721097

[409] Baxter KE Kochar S Williams C Blackman C Himmelvo J. Development of a Palliative Telehealth Pilot to Meet the Needs of the Nursing Home Population. J Hosp Palliat Nurs (2021) 23(5):478–83. 10.1097/NJH.0000000000000784 34225341

[410] Khosravi P Ghapanchi AH. Investigating the Effectiveness of Technologies Applied to Assist Seniors: A Systematic Literature Review. Int J Med Inform (2015) 85(1):17–26. 10.1016/j.ijmedinf.2015.05.014 26216463

[411] Van Den BN Schumann M Kraft K Hoffmann W. Telemedicine and Telecare for Older Patients - A Systematic Review. Maturitas (2012) 73:94–114. 10.1016/j.maturitas.2012.06.010 22809497

[412] Kim H Jung YI Kim GS Choi H Park YH. Effectiveness of a Technology-Enhanced Integrated Care Model for Frail Older People: A Stepped-Wedge Cluster Randomized Trial in Nursing Homes. Gerontologist (2021) 61(3):460–9. 10.1093/geront/gnaa090 32668005 PMC8355475

[413] Wass S Vimarlund V. The Role of ICT in Home Care. In: Studies in Health Technology and Informatics. IOS Press BV (2017). p. 153–8. 10.3233/978-1-61499-794-8-153 28809199

[414] Marcelino I Laza R Domingues P Gómez-Meire S Fdez-Riverola F Pereira A. Active and Assisted Living Ecosystem for the Elderly. Sensors (Switzerland) (2018) 18(4):1246. 10.3390/s18041246 29673234 PMC5948742

[415] Linn N Goetzinger C Regnaux JP Schmitz S Dessenne C Fagherazzi G Digital Health Interventions Among People Living With Frailty: A Scoping Review. J Am Med Directors Assoc (2021) 22:1802–12.e21. 10.1016/j.jamda.2021.04.012 34000266

[416] Calvillo‐arbizu J Naranjo‐hernández D Barbarov‐rostán G Talaminos‐barroso A Roa‐romero LM Reina‐tosina J. A Sensor‐based Mhealth Platform for Remote Monitoring and Intervention of Frailty Patients at Home. Int J Environ Res Public Health (2021) 18(21):11730. 10.3390/ijerph182111730 34770244 PMC8583636

[417] Bon V Ghemame M Fantou P Philliponnet A Mouriaux F. Feedback on Ophthalmologic Telemedicine in a Nursing Home. J Fr Ophtalmol (2020) 43(9):e293–7. 10.1016/j.jfo.2020.09.002 32977979

[418] Jaana M Paré G. Comparison of Mobile Health Technology Use for Self-Tracking Between Older Adults and the General Adult Population in Canada: Cross-Sectional Survey. JMIR Mhealth Uhealth (2020) 8(11):e24718. 10.2196/24718 33104517 PMC7717921

[419] Lee S Yu S. Effectiveness of Information and Communication Technology (Ict) Interventions in Elderly’s Sleep Disturbances: A Systematic Review and Meta-Analysis. Sensors (2021) 21:6003. 10.3390/s21186003 34577212 PMC8468949

[420] Glomsås HS Knutsen IR Fossum M Halvorsen K. They Just Came With the Medication Dispenser-a Qualitative Study of Elderly Service Users’ Involvement and Welfare Technology in Public Home Care Services. BMC Health Serv Res (2021) 21(1):245. 10.1186/s12913-021-06243-4 33740974 PMC7977566

[421] Kruse CS Mileski M Moreno J. Mobile Health Solutions for the Aging Population: A Systematic Narrative Analysis. J Telemed Telecare (2017) 23(4):439–51. 10.1177/1357633X16649790 27255207

[422] Lapão LV Peyroteo M Maia M Seixas J Gregório J Mira da Silva M Implementation of Digital Monitoring Services During the COVID-19 Pandemic for Patients With Chronic Diseases: Design Science Approach. J Med Internet Res (2021) 23(8):e24181. 10.2196/24181 34313591 PMC8396539

[423] Lang C Roessler M Schmitt J Bergmann A Holthoff-Detto V. Health-Related Quality of Life in Elderly, Multimorbid Individuals With and Without Depression And/or Mild Cognitive Impairment Using a Telemonitoring Application. Qual Life Res (2021) 30(10):2829–41. 10.1007/s11136-021-02848-8 33983617 PMC8481145

[424] Joosen P Piette D Buekers J Taelman J Berckmans D De Boever P. A Smartphone-Based Solution to Monitor Daily Physical Activity in a Care Home. J Telemed Telecare (2019) 25(10):611–22. 10.1177/1357633X18790170 30068250

[425] Andrès E Zulfiqar AA Talha S Hajjam M Hajjam J Ervé S Télémédecine et Insuffisance Cardiaque du Sujet âgé, 16. Geriatrie et Psychologie Neuropsychiatrie du Vieillissement (2018). p. 341–8. NLM (Medline). 10.1089/dia.2009.0102 30378552

[426] Liu L Stroulia E Nikolaidis I Miguel-Cruz A Rios Rincon A. Smart Homes and Home Health Monitoring Technologies for Older Adults: A Systematic Review. Int J Med Inform (2016) 91:44–59. 10.1016/j.ijmedinf.2016.04.007 27185508

[427] Majumder S Aghayi E Noferesti M Memarzadeh-Tehran H Mondal T Pang Z Smart Homes for Elderly Healthcare—Recent Advances and Research Challenges. Sensors (Switzerland) (2017) 17:2496. 10.3390/s17112496 29088123 PMC5712846

[428] Alsaqer M. Aging and Technology: Understanding the Issues and Creating a Base for Technology Designers. J Med Eng Technol (2021) 45(4):258–83. 10.1080/03091902.2021.1891313 33847223

[429] Chelongar K Ajami S. Using Active Information and Communication Technology for Elderly Homecare Services: A Scoping Review. Home Health Care Serv Q (2021) 40:93–104. 10.1080/01621424.2020.1826381 32990180

[430] Gokalp H De Folter J Verma V Fursse J Jones R Clarke M. Integrated Telehealth and Telecare for Monitoring Frail Elderly With Chronic Disease. Telemed J E Health (2018) 24:940–57. 10.1089/tmj.2017.0322 30129884 PMC6299847

[431] Sekhon H Sekhon K Launay C Afililo M Innocente N Vahia I Telemedicine and the Rural Dementia Population: A Systematic Review. Maturitas (2021) 143:105–14. 10.1016/j.maturitas.2020.09.001 33308615

[432] Brooks SE Burruss SK Mukherjee K. Suicide in the Elderly: A Multidisciplinary Approach to Prevention. Clin Geriatr Med (2019) 35:133–45. 10.1016/j.cger.2018.08.012 30390980

[433] Chen YRR Schulz PJ. The Effect of Information Communication Technology Interventions on Reducing Social Isolation in the Elderly: A Systematic Review. J Med Internet Res (2016) 18(1):e18. 10.2196/jmir.4596 26822073 PMC4751336

[434] WHO Global Observatory for eHealth. Global Diffusion of eHealth: Making Universal Health Coverage Achievable. Rep third Glob Surv eHealth (2016).

[435] World Bank. Data Blog. New World Bank Country Classifications by Income Level: 2022-2023 (2022). Available from: https://blogs.worldbank.org/en/opendata/new-world-bank-country-classifications-income-level-2022-2023 (Accessed May 9, 2024).

[436] National Institutes of Health. Aging in Place: Growing Older at Home. Natl Inst Aging (2023). Available from: https://www.nia.nih.gov/health/aging-place/aging-place-growing-older-home (Accessed July 4, 2024).

[437] Ratnayake M Lukas S Brathwaite S Neave J Henry H. Aging in Place: Are We Prepared? Dela J Public Health (2022) 8(3):28–31. 10.32481/djph.2022.08.007 36177171 PMC9495472

[438] Mace RA Mattos MK Vranceanu AM. Older Adults Can Use Technology: Why Healthcare Professionals Must Overcome Ageism in Digital Health. Transl Behav Med (2022) 12(12):1102–5. 10.1093/tbm/ibac070 36073770 PMC9494377

[439] de Siqueira Silva Í de Araújo AJ Lopes RH Silva CRDV Xavier PB de Figueirêdo RC Digital Home Care Interventions and Quality of Primary Care for Older Adults: A Scoping Review. BMC Geriatr (2024) 24(1):507–19. 10.1186/s12877-024-05120-z 38858634 PMC11163791

[440] Tsai YIP Beh J Ganderton C Pranata A. Digital Interventions for Healthy Ageing and Cognitive Health in Older Adults: A Systematic Review of Mixed Method Studies and Meta-Analysis. BMC Geriatr (2024) 24(1):217. 10.1186/s12877-023-04617-3 38438870 PMC10910826

[441] Alruwaili MM Shaban M Elsayed Ramadan OM. Digital Health Interventions for Promoting Healthy Aging: A Systematic Review of Adoption Patterns, Efficacy, and User Experience. Sustainability (Switzerland) (2023) 15(23):16503. 10.3390/su152316503

[442] Lee SH Chon Y Kim YY. Comparative Analysis of Long-Term Care in OECD Countries: Focusing on Long-Term Care Financing Type. Healthcare (2023) 11(2):206. 10.3390/healthcare11020206 36673574 PMC9858923

[443] Mounier-Jack S Mayhew SH Mays N. Integrated Care: Learning Between High-Income, and Low- and Middle-Income Country Health Systems. Health Policy Plan (2017) 32(Suppl. 4):iv6–iv12. 10.1093/heapol/czx039 29194541 PMC5886259

[444] Fang F Yang X. Socioeconomic Status, Cultural Values, and Elderly Care: An Examination of Elderly Care Preference in OECD Countries. Aging Health Res (2023) 3(3):100153. 10.1016/j.ahr.2023.100153

[445] Huenchuan S. Envejecimiento, Personas Mayores y Agenda 2030 para el Desarrollo Sostenible. Perspectiva regional y de Derechos Humanos. Desarrollo Social. Santiago: Naciones Unidas (2018). Available from: www.cepal.org/es/suscripciones (Accessed July 8, 2024).

[446] Mlinarić A Horvat M Vš S. Dealing With the Positive Publication Bias: Why You Should Really Publish Your Negative Results. Biochem Med (Zagreb) (2017) 27(3):30201. 10.11613/BM.2017.030201 PMC5696751 29180912

