# Corrigendum: Navigating Through Innovation in Elderly’s Health: A Scoping Review of Digital Health Interventions

**DOI:** 10.3389/phrs.2026.1609579

**Published:** 2026-02-17

**Authors:** Macarena Hirmas-Adauy, Carla Castillo-Laborde, Camila Awad, Anita Jasmen, Maurizio Mattoli, Xaviera Molina, Andrea Olea, Isabel Matute, Fernando Soto, Paola Rubilar, Oscar Urrejola, Tania Alfaro, María Teresa Abusleme Lama, Sophie Esnouf

**Affiliations:** 1 Centro de Epidemiología y Políticas de Salud, Facultad de Medicina Clínica Alemana, Universidad del Desarrollo, Santiago, Chile; 2 Independent Research Consulting, Santiago, Chile; 3 Centro de Informática Biomédica, Instituto de Ciencias e Innovación en Medicina, Facultad de Medicina Clínica Alemana, Universidad del Desarrollo, Santiago, Chile; 4 Escuela de Kinesiología, Facultad de Medicina Clínica Alemana, Universidad del Desarrollo, Santiago, Chile; 5 Instituto de Salud Poblacional, Facultad de Medicina, Universidad de Chile, Santiago, Chile; 6 Unidad de Salud Pública y Bioetica, Departamento de Formación Transversal en Salud, Facultad de Medicina y Ciencias de la Salud, Universidad Central de Chile, Santiago, Chile

**Keywords:** caregiver, digital health, elderly, reviews, telemedicine

One data was unintentionally omitted in Table 2 “Summary of characteristics of included documents according to PCC model (Population, Context, Concept) (Worldwide, 2022)” as published.

**TABLE 2:** Summary of characteristics of included documents according to PCC model (Population, Context, Concept) (Worldwide, 2022).

Characteristic of included documents (n = 421)		
Characteristic	N	%
Population		
Elderly	393	93,3
Healthcare professionals	202	48,0
Caregivers (n = 139)		
Family/Informal	73	52,5
Formal	52	37,4
Not specified	54	38,8
Disease or condition		
Chronic disease (hypertension, diabetes, cancer, obesity, chronic obstructive pulmonary disease, chronic pain, musculoskeletal, etc.)	218	51,8
Fragility (low grip strength, falls, balance disorders, etc.)	114	27,1
Neurological (Alzheimer’s, dementia, etc.)	101	24,0
Mental health (depression, anxiety, social isolation, loneliness, etc.)	75	17,8
Healthy ageing and quality of life	55	13,1
Other^a^	21	5,0
Sensory impairment	8	1,9
Infectious disease	6	1,4
Context		
Region of country (world Bank Region)		
Europe and central Asia	149	35,4
North America	114	27,1
East Asia and Pacific	78	18,5
Other^b^	22	5,2
Middle East and North Africa	6	1,4
Latin America and the caribbean	5	1,2
Sub-Saharan Africa	3	0,7
South Asia	1	0,2
Worldwide	39	9,3
Not specified	4	1,0
Income of country (world Bank Income)		
High	337	80,0
Upper-middle	28	6,7
Lower-middle	4	1,0
Low	1	0,2
Other^c^	8	1,9
Worldwide	39	9,3
Not specified	4	1,0
Setting		
Home	320	76,0
Nursing home (Retirement home, community care Veterans’ home, Senior living centers, Day care)	120	28,5
Healthcare facility	111	26,4
Other^d^	13	3,1
Healthcare level (n = 111)		
Primary	71	64,0
Secondary	61	55,0
Tertiary	53	47,7
Concept		
Health objective of the digital tool		
Monitoring or follow-up	265	62,9
Prevention	201	47,7
Therapy or treatment	200	47,5
Promotion	86	20,4
Diagnosis	80	19,0
Rehabilitation	70	16,6
Digital health interventions for persons		
Targeted communication to Persons	259	61,5
Personal health tracking	190	45,1
On demand communication with persons	97	23,0
Person to Person communication	81	19,2
Other^e^	38	9,0
Digital health interventions for healthcare providers		
Telemedicine	239	56,8
Healthcare provider decision support	200	47,5
Prescription and medication management	113	26,8
Referral coordination	78	18,5
Other^f^	13	3,1
Healthcare technologies results		
Positive	366	86,9
No differences	36	8,6
Partially positive	11	2,6
Negative	8	1,9
Type of digital health tools		
Hardware or physical devices		
Telephone (cell phone, smart phone, landline phone)	216	51,3
Desktop or laptop computer	166	39,4
Wearable activity monitors (wrist-worn devices, or step counters)	98	23,3
Tablet	91	21,6
Sensors and positioning system	89	21,1
Video game consoles, exergames, balance board, dance mat, handheld remotes, fitness board, buzz controller, force platforms, cameras with gesture recognition, virtual environment non-immersive and inmersive	55	13,1
Telehealth devices (with or without health measurement)	50	11,9
Other^g^	37	8,8
Interactive TV	34	8,1
Robots, social robots, robotic rollators, industrial and service robots, assistive telepresence robot	11	2,6
Radio RX/TX with interaction	2	0,5
Software, platforms		
Videoconference platforms	153	36,3
Text/Audio messaging (SMS, chat, etc.)	110	26,1
Digital health portals	91	21,6
Health and fitness apps	81	19,2
Video game	63	15,0
Electronic mail	50	11,9
Digital community or groups	35	8,3
Other^h^	34	8,1

In this table, next to the word caregivers, there should be a parenthesis with (n = 139). As it appears in healthcare level which has (n = 111).

In addition, in the percentage’s column, the data of Family/Informal, Formal and Not specified should be modified by replacing:

- 17.3 with 52.5 in Family/Informal
- 12.4 with 37.4 in Formal
- 12.8 with 38.8 in Not specified

The corrected Table 2 appears below.

In the first published version of the article, on page 5, the % of caregivers was calculated using a different criterion.

A correction has been made to **Results**, Population, Paragraph 1. The correct paragraph is: “The interventions were primarily aimed at the elderly population (93.3%, n = 393), followed by healthcare professionals (48.0%, n = 202), and caregivers (33.0%, n = 139). Among the interventions focused on caregivers, the most studied group were family or informal caregivers (52.5%, n = 73) (Table 2).”

The authors apologize for this error and state that this does not change the scientific conclusions of the article in any way. The original article has been updated.

